# Supplementary material for: Beta Cell Hubs Dictate Pancreatic Islet Responses to Glucose
Source: Cell Metab. 2016 Sep 13;24(3):389–401. doi: 10.1016/j.cmet.2016.06.020 (PMC5031557; doi:10.1016/j.cmet.2016.06.020)
Supplement: Document S1. Figures S1–S7 and Supplemental Experimental Procedures [file mmc1.pdf]

Cell Metabolism, Volume *24*

Supplemental Information

Beta Cell Hubs Dictate

Pancreatic Islet Responses to Glucose

Natalie R. Johnston, Ryan K. Mitchell, Elizabeth Haythorne, Maria Paiva Pessoa, Francesca Semplici, Jorge Ferrer, Lorenzo Piemonti, Piero Marchetti, Marco Bugliani, Domenico Bosco, Ekaterine Berishvili, Philip Duncanson, Michael Watkinson, Johannes Broichhagen, Dirk Trauner, Guy A. Rutter, and David J. Hodson

SUPPLEMENTAL FIGURES

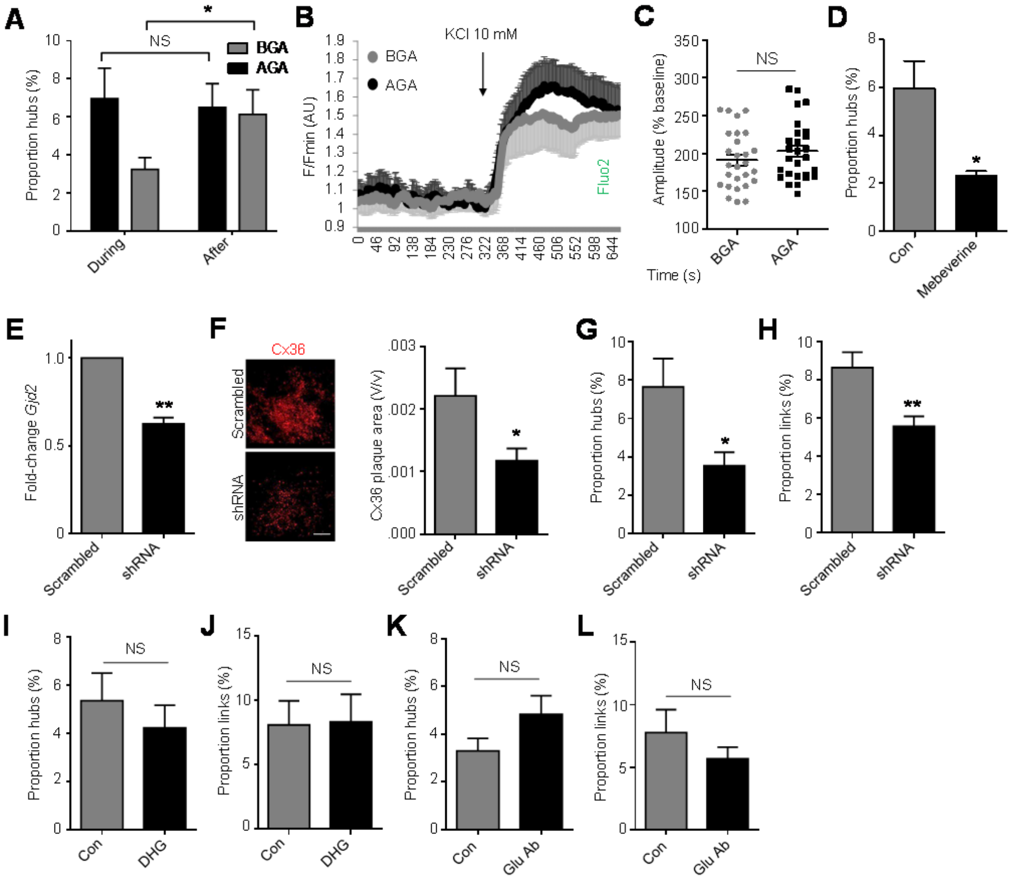

Figure S1. Gap junction inhibition with AGA is reversible and glucagon does not influence hub function. Related to Figure 1.

(A) The effects of 18-α-glycyrrhetinic acid (AGA; 20 µM) on hub number are reversible following washout (control is glycyrrhizic acid; BGA).

(B) Mean traces from a representative recording showing that AGA does not impair KCl-induced Ca^{2+} rises in dissociated single β cells *versus* BGA (20 µM) (*n* = 11 cells).

(C) As for (B), but summary data for all experiments (*n* = 27 cells from 3 animals).

(D) Mebeverine, another GJ blocker with a different mode of action, exerts similar effects to AGA (*n* = 5 recordings from 3 animals).

(E) Adenovirus harboring shRNA against *Gjd2* reduces expression by ~ 40% in whole islets (expected to be even higher at the surface) (*n* = 5 recordings from 6 animals) (scale bar, 25 µm).

(F) As for (E), but immunohistochemistry showing a large reduction in connexin-36 (Cx36) protein abundance following treatment with *Gjd2* shRNA (*n* = 7 islets from 3 animals).

(G-H) *Gjd2* knockdown decreases proportion of hubs (G) and correlated links (H) in mouse islets (*n* = 8 recordings from 6 animals).

(I-J) Hub function (I) and correlated population activity (J) is unaffected in the presence of 1 µM des-His^1-$[Glu^9]$-Glucagon (1-29) amide (DHG), a glucagon receptor antagonist (*n* = 6 recordings from 6 animals).

(K-L) As for (I-J), but in the presence of a mouse anti-glucagon antibody 1:1000 (*n* = 6 recordings from 6 animals).

Data are means ± SEM. **P<0.01, *P<0.05 and NS, non-significant.

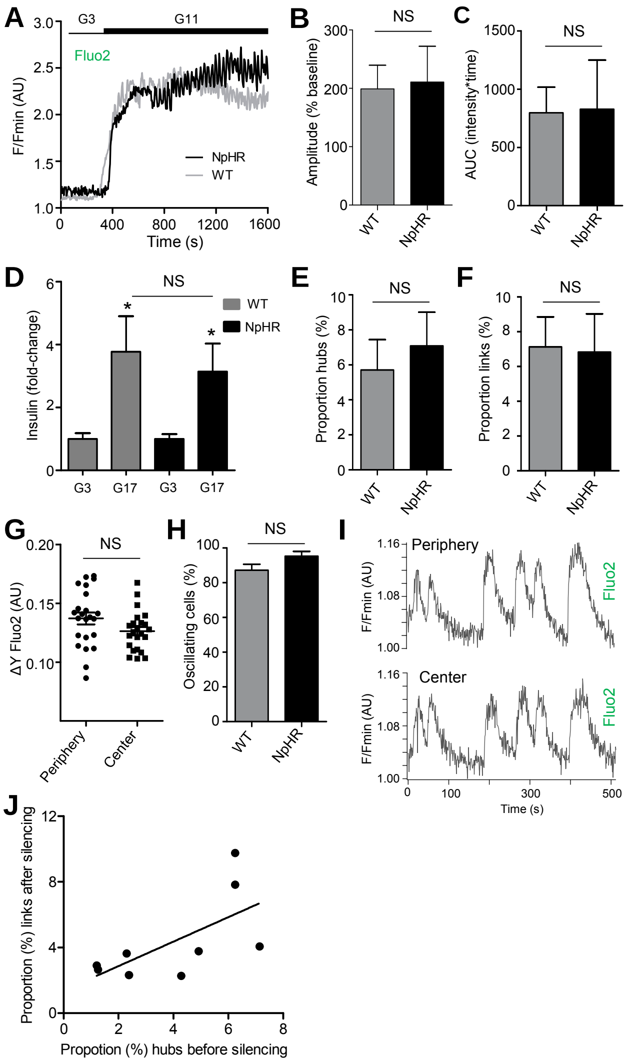

Figure S2. Ionic fluxes and insulin secretion in eNpHR3.0 islets, and hub number-silencing strength relationships. Related to Figure 4 and Figure 5.

(A) Glucose-stimulated intracellular Ca^{2+} rises are identical in wild-type (WT) and eNpHR3.0-expressing (NpHR) islets (mean of *n* = 6 recordings).

(B) Bar graph showing similar amplitude Ca^{2+} rises in WT and eNpHR3.0-expressing islets (*n* = 6 recordings).

(C) As for (B), but AUC.

(D) Glucose-stimulated insulin secretion *in vitro* is similar in WT and NpHR islets (NS, non-significant *versus* WT) (*n* = 4 animals).

(E) The number of hubs as a proportion of the imaged population is similar in WT and NpHR islets (*n* = 6 recordings).

(F) The proportion of correlated links (*i.e.* coordinated activity level) is similar in WT and NpHR islets (*n* = 6 recordings).

(G) No difference in the amplitude of Ca^{2+} rises is detected in cells located at the periphery or center of NpHR islets (*n* = 4 recordings).

(H) The proportion of cells displaying oscillatory behavior is similar in WT and NpHR islets (*n* = 6 recordings).

(I) Representative Ca^{2+} traces from the periphery and center of an NpHR islet.

(J) The strength of inhibition of islet Ca^{2+} responses (proportion (%) links) following targeting of an individual hub tends to be inversely correlated with the total number of these cells per islet before silencing (linear regression; R^2 = 0.42 and P<0.0601) (*n* = 9 recordings from 4 animals). That is, the non-silenced hubs can maintain some coordinated responsiveness.

For A-H, the signal attributable to membrane-bound EYFP was subtracted to allow more accurate comparison. Data are means ± SEM. *$P<0.05$ and NS, non-significant.

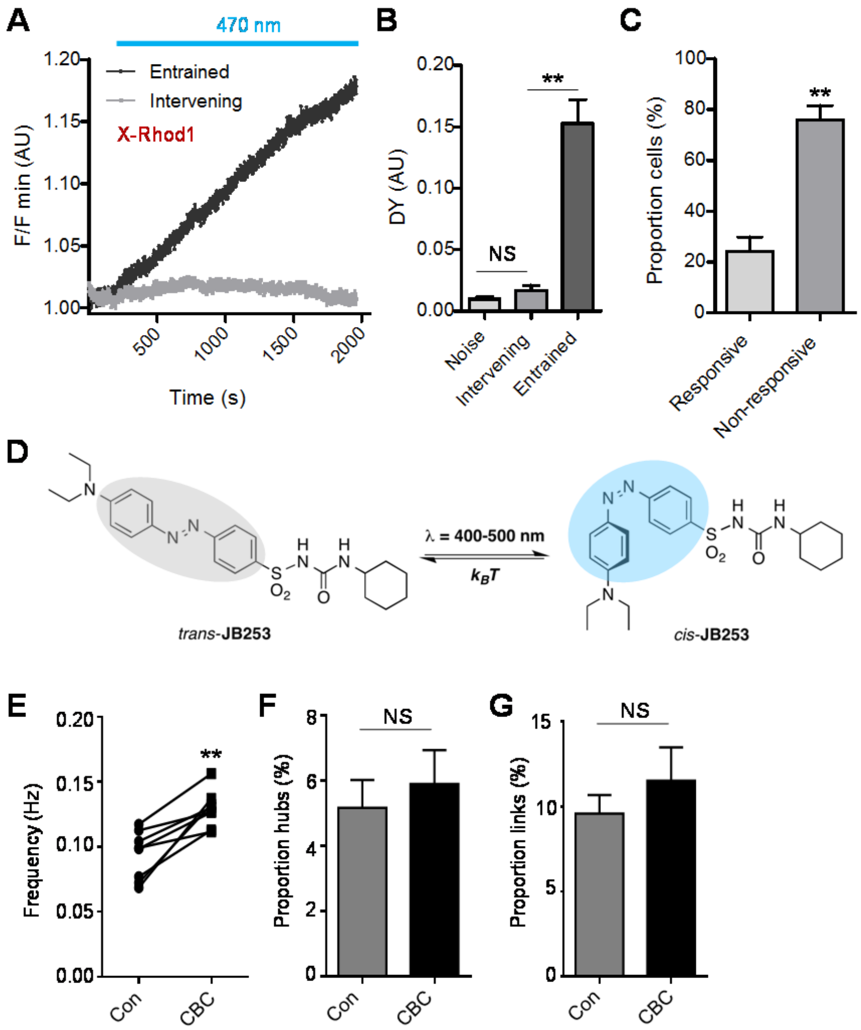

Figure S3. Photoactivated JB253 does not diffuse in the tissue and carbachol increases population frequency. Related to Figure 5.

(A) Mean intensity-over-time traces for X-Rhod1 in entrained and intervening (*i.e.* those located between the hub and follower) cells following targeted stimulation of a JB253-treated islet (*n* = 6 recordings from 4 animals).

(B) As for (A), but bar graph showing amplitude (DY) of Ca^{2+} rises in intervening and entrained cells.

(C) Only 20% of cells respond to targeted stimulation with Ca^{2+} rises, consistent with the number of followers linked to a hub (*n* = 6 recordings from 4 animals).

(D) Chemical structure of JB253 showing the *trans* (dark)- and *cis* (blue light)-isomers, the former possessing some activity in the dark due its photostationary state.

(E-G) The acetylcholinomimetic carbachol (CBC; 10 μM) increases population Ca^{2+}-spiking frequency (E), without altering the proportion of either hubs (F) or correlated links (G).

Data are means ± SEM. **P<0.01 and NS, non-significant.

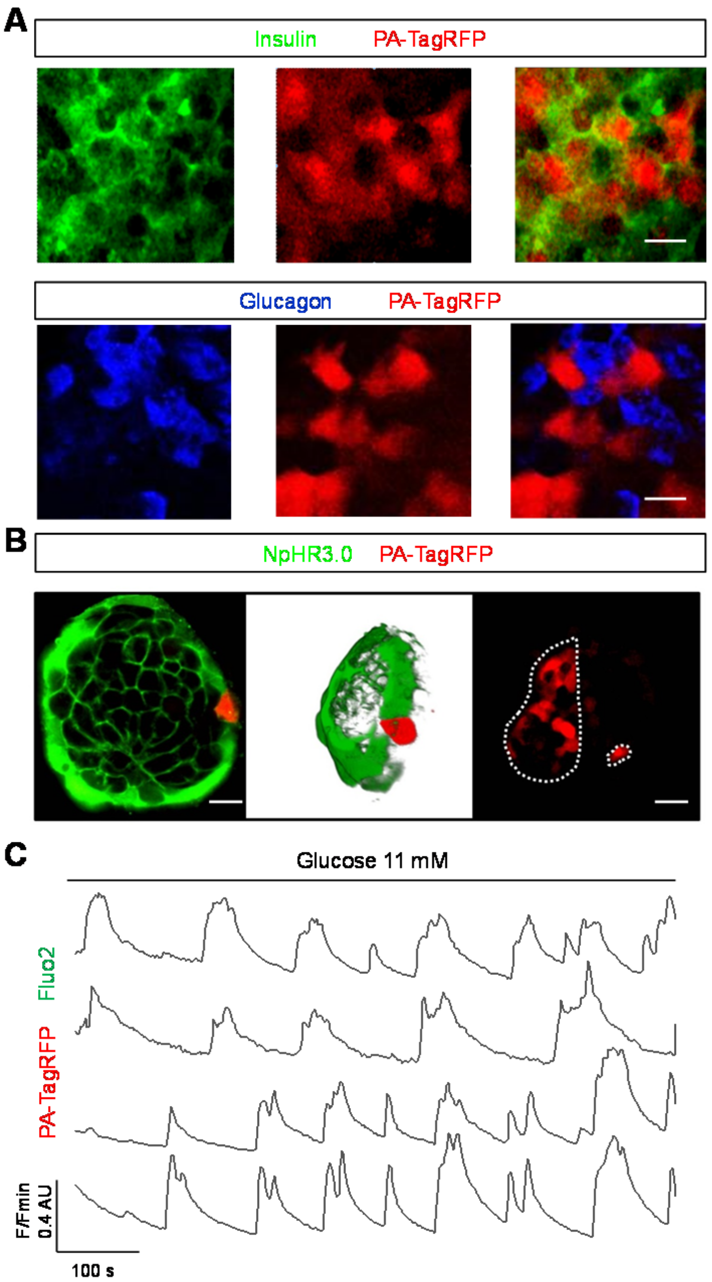

Figure S4. Single cell photoactivation of PA-TagRFP in intact islets. Related to Figure 6.

(A) PA-TagRFP is specifically expressed in β- but not α-cells, as assessed using immunostaining for insulin and glucagon, respectively (scale bar = 12.5 µm).

(B) Left panel: a single photopainted cell in a PA-TagRFP-expressing and fluo2-loaded islet ~ 40 µm from the surface (left) (63 x/1.4 NA objective). Middle panel: 3D opacity render of the same sample to show a single highlighted cell. Right panel: a single cell was targeted followed by half the islet (20x /0.8NA objective) (scale bar = 20 µm).

(C) Ca^{2+} oscillations are not perturbed by adenoviral-introduction of PA-TagRFP into islets (*n* = 3 recordings).

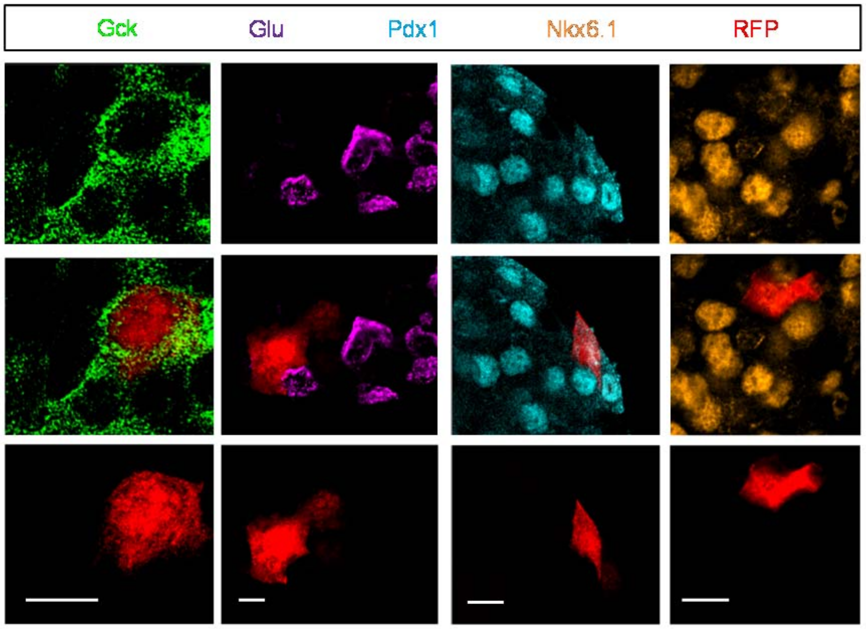

Figure S5. High-resolution snapshots of photopainted hubs. Related to Figure 6.

Hubs (RFP; red) express more glucokinase (Gck), less Pdx1 and less Nkx6.1 *versus* the rest of the population. No glucagon (Glu) expression is evident. Scale bar = 12.5 µm. Images captured in the intact islet preparation (*n* = 5-9 hubs from 3-4 animals) using a Zeiss LSM780 confocal microscope (63x/1.4NA objective).

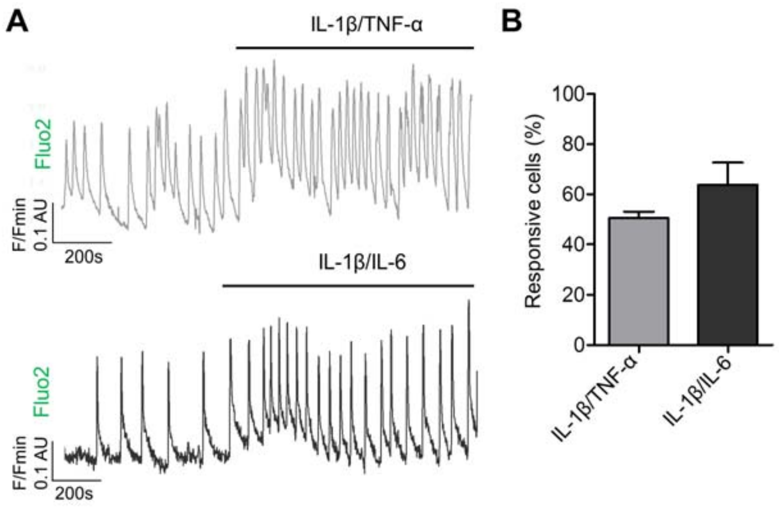

Figure S6. Acute effects of cytokine on β cell ionic fluxes. Related to Figure 7.

(A) Representative traces showing that application of either IL-1β/TNF-α or IL-1 β/IL-6 acutely upregulates Ca^{2+} spiking activity in islets.

(B) Bar graph showing that over half of the β cell population responds to acute cytokine exposure (*n* = 7-8 recordings from 4 animals) (186/368 cells for IL-1β/TNF-α; 174/279 cells for IL-1 β/IL-6).

Data are means ± SEM.

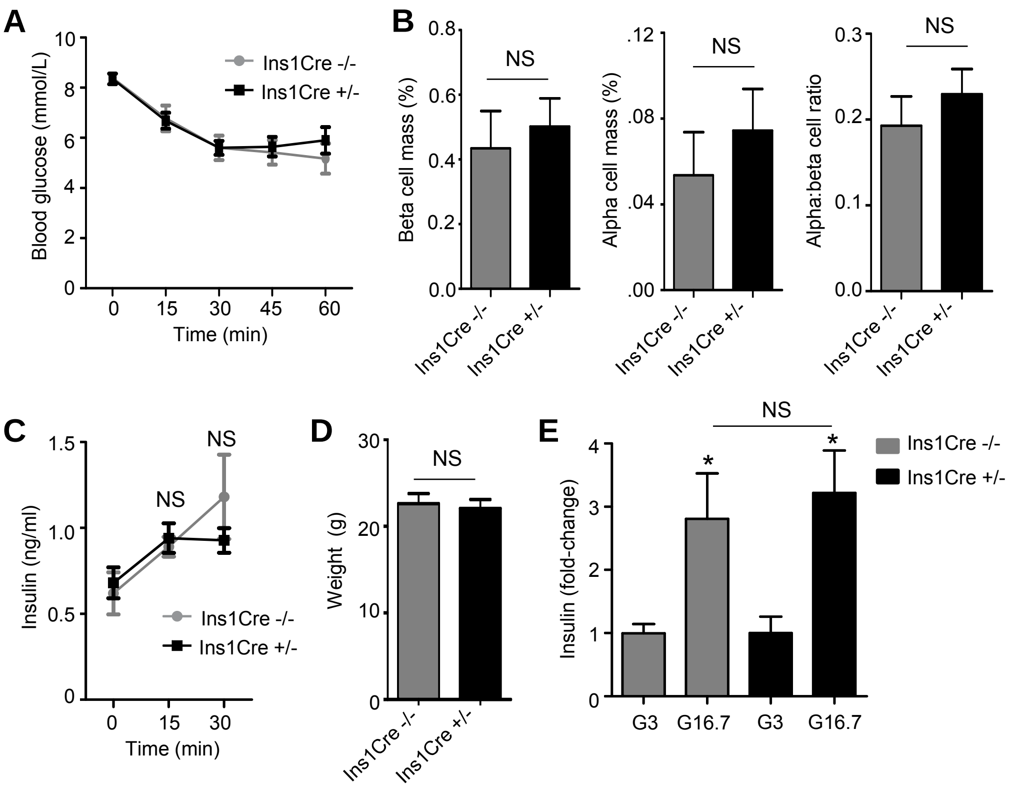

Figure S7. Phenotyping of Ins1Cre animals. Related to the Experimental Procedures.

(A) *Ins1Cre*$^{-/-}$ and *Ins1Cre*$^{+/-}$ animals display similar insulin tolerance (*n* = 4-6).

(B) β cell and alpha cell mass, as well as the α:β cell ratio are not significantly different in Ins1Cre$^{-/-}$ and Ins1Cre$^{+/-}$ animals (*n* = 3).

(C) *In vivo* insulin secretion is similar in Ins1Cre$^{-/-}$ and Ins1Cre$^{+/-}$ mice (*n* = 4-6).

(D) Body weights are similar in Ins1Cre$^{-/-}$ and Ins1Cre$^{+/-}$ mice (*n* = 4-6).

(E) Glucose-stimulated insulin secretion *in vitro* is similar in Ins1Cre$^{-/-}$ and Ins1Cre$^{+/-}$ islets (NS, non-significant *versus Ins1Cre*$^{-/-}$) (*n* = 4 animals).

Data are means ± SEM. *P<0.05 and NS, non-significant.

SUPPLEMENTAL MOVIE LEGENDS

Movie S1. Dynamics of hub function. Magnified timelapse recording of Ca^{2+} response in a wild-type islet showing a hub (circled in yellow) repetitively firing before the rest of the population. Islet was incubated in 11 mM glucose. Note the longer duty cycle. Playback rate 40 frames per second (fps). Related to Figure 1.

Movie S2. Optogenetic silencing of β cell function. Timelapse recording of Ca^{2+} responses in eNpHR3.0-expressing islets subjected to repeated silencing using a 561nm global laser (10 x objective). A Yokogawa Nipkow spinning disk head was used to rapidly scan a large field of view without introducing lag artefacts. A stable baseline from which to discriminate silencing from endogenous oscillatory activity was achieved by incubating islets in 100 µM tobultamide and 11 mM glucose. Movie has been cropped to display a single islet. Related to Figure 2.

Movie S3. Optogenetic targeting of β cells in a single islet. Timelapse recording of Ca^{2+} responses in two eNpHR3.0-expressing islets subjected to global silencing (left and right), before targeting of a single islet (right) (10 x objective). A stable baseline from which to discriminate silencing from endogenous oscillatory activity was achieved by incubating islets in 100 µM tobultamide and 11 mM glucose. Playback rate 40 frames per second (fps). Related to Figure 2.

Movie S4. Optogenetic targeting of β cells in an islet subregion. Timelapse recording of Ca^{2+} responses in an NpHR3.0-expressing islet subjected to global silencing (x1), before targeting (x2) of a cell triplet (right) (10 x objective). A stable baseline from which to discriminate silencing from endogenous oscillatory activity was achieved by incubating islets in 100 µM tobultamide and 11 mM glucose. Playback rate 40 frames per second (fps). Movie has been cropped to display a single islet. Related to Figure 2.

Movie S5: β cell population responses before hub silencing. $Beta$ cell population Ca2+ dynamics in eNpHR3.0-expressing islet before optogenetic silencing of a hub cell (20 x objective). Islet was incubated in 11 mM glucose. Playback rate 40 frames per second (fps). Related to Figure 5.

Movie S6: β cell population responses during hub silencing. Same islet as in Movie S5, but showing β cell population Ca2+ dynamics during optogenetic silencing of a hub cell (20 x objective). Islet was incubated in 11 mM glucose. Playback rate 40 frames per second (fps). Related to Figure 5.

SUPPLEMENTAL EXPERIMENTAL PROCEDURES

Animals, glucose/insulin tolerance testing and insulin measures

Mice were maintained in a specific pathogen-free (SPF) facility under a 12 h light–dark cycle with *ad libitum* access to water and standard laboratory diet. One copy of the floxed allele was sufficient to drive widespread eNpHR3.0-EYFP expression in β cells under the powerful ROSA26-targeted CAG promoter (*i.e. Ins1Cre*$^{+/-}$*;eNpHR3.0-EYFP*$^{fl/-}$, derived from an *Ins1Cre*$^{-/-}$*;eNpHR3.0-EYFP*$^{fl/fl}$ x *Ins1Cre*$^{+/-}$*;eNpHR3.0-EYFP*$^{-/-}$ breeding pair). Cre-mediated recombination efficiency was 94.3 ± 1.9 % in our hands, similar to that shown already (Thorens et al., 2014). To account for any effects that loss of an insulin allele may have on glucose tolerance, experiments were also performed in mice where eNpHR3.0 is activated on an Ins1Cre background (*i.e.* Ins1Cre$^{+/-}$;eNpHR3.0$^{fl/-}$ and *Ins1Cre*$^{+/-}$*;eNpHR3.0*$^{-/-}$ animals). Glucose and insulin tolerance tests were conducted using established protocols (Lemaire et al., 2009). Plasma insulin levels were determined at 0, 15 and 30 min following intraperitoneal injection of glucose (3 g/kg) using a Crystal Chem ultrasensitive ELISA. All procedures were regulated by the Home Office according to the Animals (Scientific Procedures) Act 1986 of the United Kingdom (PPL 70/7349).

Electrophysiology

β cells were identified morphologically and by depolarization of the membrane potential in response to 16.7 mM glucose. Extracellular solution was HEPES-buffered saline containing in mM: 135 NaCl, 5 KCl, 1 $MgCl_2$, 1 $CaCl_2$, 10 HEPES and 16.7 *D*-glucose (pH 7.4). For perforated patch, recording electrodes (5-10 MΩ resistance; borosilicate glass) were filled with a solution containing in mM: 140 KCl, 5 $MgCl_2$, 3.8 $CaCl_2$, 10 HEPES, 10 EGTA (pH 7.2) and 20–25 µg/ml amphotericin B (Beall et al., 2010). For whole-cell experiments, recording electrodes (5-10 MΩ resistance; borosilicate glass) were filled with a solution containing in mM: 140 KCl, 5 MgCl2, 3.8 $CaCl_2$, 10 HEPES, 10 EGTA (pH 7.2) and 3 ATP (Beall et al., 2010). These internal solutions contain similar levels of Cl^- to those used previously in β cells (Dadi et al., 2014) and for the study of eNpHR3.0 (Alfonsa et al., 2015; Song et al., 2013), and avoid liquid junction potential while maximising β cell action potential firing (Best, 2005). In perforated patch-clamp experiments, the resting membrane potential was recorded for 3 min before yellow light (λ = 572 nm) exposure for 3-5 min. In the voltage-clamp whole cell experiments, current was recorded in response to a 1 s pulse of yellow light.

Calcium and mitochondrial potential imaging

Islets were loaded with Fluo2 (Teflab) for 30-45 min before rapid capture of Ca^{2+} signals with cell resolution using a Yokogawa CSU22 Nipkow spinning disk coupled to a Zeiss Axiovert M200 and x10–x20/0.3–0.8NA objectives (Zeiss). By calculating the point-spread-function (PSF) of PS-Speck beads (100 nm; Invitrogen) for a 20x objective, this gives lateral and axial resolutions of 0.65 +/- 0.23 µm and 3.56 +/- 0.76 µm, respectively. Fluo2 was excited at λ = 491 nm using a DPSS laser (Cobalt) and emission detected at λ = 525/25 nm using a 16-bit EM-CCD (Hammamatsu). Ca^{2+} imaging using GCaMP6m was performed identically, except following 48 hr incubation with adenovirus (1.3 x 10^8 PFU/islet) harboring the cDNA for the genetically-encoded Ca^{2+} indicator (Vector Biolabs). Throughout, islets were maintained at 34-36 ^{0}C in a stainless steel chamber (Attofluor) and irrigated with HEPES-bicarbonate buffer containing in mM: 120 NaCl, 4.8 KCl, 24 $NaHCO_3$, 0.5 Na_2HPO_4, 5 HEPES, 2.5 $CaCl_2$, 1.2 $MgCl_2$ and 11 *D*-glucose. Single cell silencing in eNpHR3.0-expressing tissue was achieved using a λ = 589 nm laser (300 mW; CNI Laser) linked *via* a single-mode fibre optic to a custom-manufactured dichroic array, configured to deliver a diffraction-limited (~ 500 nm) spot (6.8-23.4 mW/mm^2) to the focal plane (Cairn Research). Single cell stimulation was performed using the photoswitchable sulfonylurea JB253 and a diffraction-limited λ = 470 nm laser.

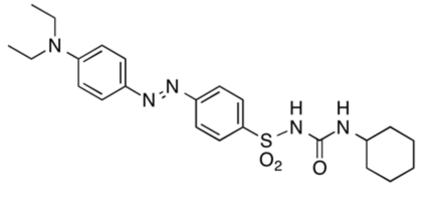

trans-**JB253**

JB253 was synthesized and used as previously described (Broichhagen et al., 2014), and combined with the Ca^{2+} indicator X-Rhod1 (excitation λ = 561 nm, emission λ = 630/75 nm) to avoid spectral overlap. JB253 occupies a photostationary state that results in ~10-20% K_{ATP} channel block (Broichhagen et al., 2014), a pre-requisite for proper propagation of signals *via* gap junctions (Zhang et al., 2008). However, following exposure to blue light, JB253 undergoes isomerization *via* its azobenzene photoresponsive unit, subtly changing molecule conformation at SUR1 to allow ~70% K_{ATP} channel closure (Broichhagen et al., 2014). Mitochondrial potential was monitored using 20 nM tetramethylrhodamine ethyl ester (TMRE) and excitation and emission at λ = 561 nm and λ = 630/75 nm, respectively (Hodson et al., 2014). Drugs were applied *via* the perfusion system at the indicated times and concentration.

Recordings were only included in subsequent analyses if: 1) the targeted cell itself responded with an increase/decrease in Ca^{2+} levels; and 2) silenced cells recovered from inhibition with resumption of Ca^{2+}-spiking activity. The overexpressed EYFP is unlikely to interfere with recordings of cytoplasmic Ca^{2+}, since the former is membrane-localized, and the amplitude of Ca^{2+} rises and AUC in response to elevated glucose was identical in wild-type and eNpHR3.0 islets, as shown in Figure S2. In all cases, care was taken to prevent artefacts due to phototoxicity/photodamage, for example by using multifocal microscopy to decrease pixel dwell time, employing highly-sensitive EM-CCD detectors, reducing laser power to the minimum required for an adequate signal-to-noise ratio (SNR), and limiting imaging duration. Experiments showing signs of phototoxicity (*i.e.* large and sustained increases/decreases in intracellular Ca^{2+} concentration, excessive photobleaching etc.) were discarded.

shRNA-silencing of connexin-36
Adenoviral particles carrying an shRNA expression construct against mouse *Gjd2* were acquired from Vector Biolabs and applied to islets at 7 x 10^9 PFU/islet for 24 hrs. Transduction was confirmed using GFP expression, as well as real-time PCR and immunohistochemistry for *Gjd2/*Cx36. In all cases, scrambled shRNA was used as control.

Immunohistochemistry
Confocal imaging was performed using a Zeiss LSM 780 Meta Confocal (x20-x63/0.8-1.4 NA objectives) configured with GaAsP spectral detectors set to collect emitted signals at 500-550 nm (Alexa Fluor-488), 561-597nm (Alexa Fluor-568) and 642-701nm (Alexa Fluor-633). The fraction area occupied by insulin, glucokinase (Gck), Pdx1, Tomm20, Nkx6.1, SERCA2 and Pdi was calculated using the threshold-corrected image (threshold plugin for ImageJ), and levels compared between photopainted cells and the remainder of the population. Mitochondrial morphology was assessed by measuring sphericity (Volocity) and length (BoneJ for Fiji) (Doube et al., 2010). To produce confocal superresolution images, the image was Nyquist-sampled in a 54 nm x 54 nm x 160 nm voxel, before iterative offline deconvolution using PSF algorithms embedded in Huygens software (Scientific Volume Imaging). The primary antibodies used were: guinea pig anti-insulin 1:200 (DAKO), mouse anti-glucagon 1:1000 (Sigma), rabbit anti-Gck 1:50 (Santa-Cruz), rabbit anti-Pdx1 1:50 (Rafiq, 2000), goat anti-Pdx1 1:2500 (Abcam), mouse anti-neurogenin 3 (Ngn3) 1:1000 (DSHB Hybridoma Product F25A1B3), rabbit anti-Tomm20 1:250 (Santa Cruz Biotechnology), rabbit anti-Nkx6.1 1:250 (Sigma), rabbit anti-SERCA2 1:250 (Alomone Labs), rabbit anti-Pdi 1:200 (Cell Signaling Technology) and rabbit anti-Cx36 1:50 (Zymed).The secondary antibodies used were Alexa Fluor-488, -568 and -633 (1:500) (Invitrogen). To live image mitochondria, islets were incubated in 100 nM MitoTracker Red FM (Invitrogen) for 30 min

β and α cell mass
β and α cell mass were measured using wax-embedded tissue slices taken at 25 μm intervals throughout the entire pancreas, and stained with guinea pig anti-insulin 1:200 (DAKO; Alexa Fluor-488 secondary) and rabbit anti-glucagon 1:100 (Santa-Cruz; Alexa Fluor-568 secondary) antibodies

Necrosis and apoptosis assays
Islets were incubated in 3 μM calcein AM (Invitrogen) and 2.5 μM propidium iodide (PI) (Sigma-Aldrich) for 15 min before detection of absorbance/emission at 491/525 nm and 561/620 nm, respectively. The islet area occupied by dead cells (PI) was expressed *versus* that occupied by live (calcein) and dead cells (V/v). Apoptosis was assessed using a DeadEnd Fluorimetric TUNEL System staining kit (Promega) according to the manufacturer's instructions and absorbance/emission detected at 491/525nm. The proportion of apoptotic β cells was expressed as a fraction area *versus* non-apoptotic cell mass (V/v).

Photopainting
Photopainting was performed *post facto* following fMCI and correlation analysis by bounding the identified cell(s) with a region of interest (ROI) and scanning the area with a λ = 405 nm diode laser (x20/0.8NA objective).

Correlation, similarity analyses and polar coordinates
Endocrine cells display Ca^{2+} spikes with complex frequencies, amplitudes and inter-event intervals, rendering algorithms based on spike detection unreliable. Thus, binarization was instead employed where cells are considered to be either 'ON' or 'OFF' at a given time point, depending on whether they are above a threshold value (20%; to account for the signal-to-noise ratio). The resulting binarized traces were subjected to correlation analyses based upon observations of cell synchrony using:

$$C_{ij} = \frac{T_{ij}}{\sqrt{T_i T_j}}$$

Where C is a correlation coefficient between -1 to +1, T_{ij} is the total time spent in the coactive state between node i and j, and T_i and T_i represent the time spent in the active phase for each individual node. To identify cell pairs correlated (*i.e.* linked) with a higher-than-chance probability, significance ($P<0.01$) of interactions was computed using a non-deterministic Monte-Carlo statistic *versus* the permuted dataset (> 9,999 iterations). Monte-Carlo analyses use computationally-intensive random sampling algorithms to model uncertainty (*i.e.* how likely are we to reproduce a given wiring patterns simply due to chance?). Functional connectivity maps were then constructed based on the distribution of correlations (or links) (k) between cells (n), allowing visualization of the network. The resulting link-probability distribution was best-fitted using the power-law function $f(x) = axk + o(xk)$ and cells occupying the 60-100% connectivity range (*i.e.* those possessing the majority of the links) defined as hubs. Power-law describes the relationship where a given variable changes proportionally to the power of the other. Such distributions are widely observed in physical and social systems and are typified by a long tail where the minority possess the majority (*e.g.* both wealth or scientific citations are power-law distributed). Goodness-of-fit was determined using a log-transformed linear regression model (R^2) and cross-checked against the non-linear minimization of the residual sum of squares (RSS) using heuristic methods (Nelder-Mead). To determine the stability of network topology, islets were considered 'before' and 'after' optogenetic silencing. The linear correlation between the resulting similarity matrices (*i.e.* how similar are the connection distribution/wiring patterns?) was then calculated using the Pearson R and significance considered against either: 1) a matrix with enforced dissimilarity (*i.e.* 'during' optogenetic silencing for each islet); or 2) a shuffled matrix (*i.e.* random permutation/shuffling for each islet). R values above and below 0 suggest positive and negative correlation, respectively. Polar coordinates (r,θ) were calculated from the Cartesian coordinates (x,y) using r (distance) = $\sqrt{(x^2 + y^2)}$ and $\theta = \tan^{-1}(y/x)$.

Measurements of insulin secretion from isolated islets

Insulin secretion was measured using static incubation of islets (batches of 6-8) for 30 min in HEPES-bicarbonate buffer at 37 °C containing the indicated glucose concentrations (da Silva Xavier et al., 2009). Insulin concentration in the supernatant was determined using a proprietary Homogeneous Time Resolved Fluorescence (HTRF)-based assay (Cisbio) according to the manufacturer's instructions.

Dynamic insulin secretion measures

Briefly, hubs were identified *a priori* using Ca^{2+} imaging before monitoring Zn^{2+} accumulation (excitation λ = 405 nm, emission λ = 450/50 nm) using an X-Light/Nikon Eclipse Ti-E spinning disk system in bypass mode and a x20/0.75 NA objective (Nikon). Cell silencing was performed with a user-defined ROI in Metamorph software and an $iLas^2$ photoactivation module (BioVision) coupled to a 585 nm laser (Coherent OBIS). Global silencing was performed using a 561 nm laser (Coherent OBIS). JP-107 is derived from click-S_NAR-click chemistry, contains a Zn^{2+}-chelating site and displays increases in fluorescence intensity due to accumulation of the ion over time (Pancholi et al., 2014). Thus, insulin secretion rate was calculated for each individual experiment by fitting a linear regression model. The mean slope value was then statistically compared for each condition examined (*i.e.* before and during laser exposure), and the P-value displayed on the corresponding graph. Negative rates are observed when insulin secretion is unable to surpass net Zn^{2+} dissolution from the probe in the presence of 1 µM EDTA (added to reduce background noise).

SUPPLEMENTAL REFERENCES

Alfonsa, H., Merricks, E.M., Codadu, N.K., Cunningham, M.O., Deisseroth, K., Racca, C., and Trevelyan, A.J. (2015). The contribution of raised intraneuronal chloride to epileptic network activity. J. Neurosci. *35*, 7715-7726.

Beall, C., Piipari, K., Al-Qassab, H., Smith, M.A., Parker, N., Carling, D., Viollet, B., Withers, D.J., and Ashford, M.L. (2010). Loss of AMP-activated protein kinase alpha2 subunit in mouse beta-cells impairs glucose-stimulated insulin secretion and inhibits their sensitivity to hypoglycaemia. Biochem. J. *429*, 323-333.

Best, L. (2005). Glucose-induced electrical activity in rat pancreatic beta-cells: dependence on intracellular chloride concentration. J. Physiol. *568*, 137-144.

Broichhagen, J., Schönberger, M., Cork, S.C., Frank, J.A., Marchetti, P., Bugliani, M., Shapiro, A.M.J., Trapp, S., Rutter, G.A., Hodson, D.J., et al. (2014). Optical control of insulin release using a photoswitchable sulfonylurea. Nat. Commun. *5*, 5116.

da Silva Xavier, G., Loder, M.K., McDonald, A., Tarasov, A.I., Carzaniga, R., Kronenberger, K., Barg, S., and Rutter, G.A. (2009). TCF7L2 regulates late events in insulin secretion from pancreatic islet beta-cells. Diabetes *58*, 894-905.

Dadi, P.K., Vierra, N.C., and Jacobson, D.A. (2014). Pancreatic beta-cell-specific ablation of TASK-1 channels augments glucose-stimulated calcium entry and insulin secretion, improving glucose tolerance. Endocrinology *155*, 3757-3768.

Doube, M., Kłosowski, M.M., Arganda-Carreras, I., Cordelières, F.P., Dougherty, R.P., Jackson, J.S., Schmid, B., Hutchinson, J.R., and Shefelbine, S.J. (2010). BoneJ: Free and extensible bone image analysis in ImageJ. Bone *47*, 1076-1079.

Hodson, D.J., Tarasov, A.I., Gimeno Brias, S., Mitchell, R.K., Johnston, N.R., Haghollahi, S., Cane, M.C., Bugliani, M., Marchetti, P., Bosco, D., et al. (2014). Incretin-modulated beta cell energetics in intact islets of Langerhans. Mol. Endocrinol. *28*, 860-871.

Lemaire, K., Ravier, M.A., Schraenen, A., Creemers, J.W., Van de Plas, R., Granvik, M., Van Lommel, L., Waelkens, E., Chimienti, F., Rutter, G.A., et al. (2009). Insulin crystallization depends on zinc transporter ZnT8 expression, but is not required for normal glucose homeostasis in mice. Proc. Natl. Acad. Sci. U. S. A. *106*, 14872-14877.

Pancholi, J., Hodson, D.J., Jobe, K., Rutter, G.A., Goldup, S.M., and Watkinson, M. (2014). Biologically targeted probes for Zn^{2+}: a diversity oriented modular “click-SNAr-click” approach. Chemical Science *5*, 3528-3535.

Rafiq, I. (2000). Glucose-stimulated Preproinsulin Gene Expression and Nuclear trans-Location of Pancreatic Duodenum Homeobox-1 Require Activation of Phosphatidylinositol 3-Kinase but Not p38 MAPK/SAPK2. J. Biol. Chem. *275*, 15977-15984.

Song, J., Sun, J., Moss, J., Wen, Z., Sun, G.J., Hsu, D., Zhong, C., Davoudi, H., Christian, K.M., Toni, N., et al. (2013). Parvalbumin interneurons mediate neuronal circuitry–neurogenesis coupling in the adult hippocampus. Nat. Neurosci. *16*, 1728-1730.

Thorens, B., Tarussio, D., Maestro, M.A., Rovira, M., Heikkila, E., and Ferrer, J. (2014). Ins1 knock-in mice for beta cell-specific gene recombination. Diabetologia *58*, 558-565.

Zhang, Q., Galvanovskis, J., Abdulkader, F., Partridge, C.J., Gopel, S.O., Eliasson, L., and Rorsman, P. (2008). Cell coupling in mouse pancreatic beta-cells measured in intact islets of Langerhans. Philos. Transact. A Math. Phys. Eng. Sci. *366*, 3503-3523.
