# Supplementary figures and images for: Beta Cell Hubs Dictate Pancreatic Islet Responses to Glucose
Source: Cell Metab. 2016 Sep 13;24(3):389–401. doi: 10.1016/j.cmet.2016.06.020 (PMC5031557; doi:10.1016/j.cmet.2016.06.020)

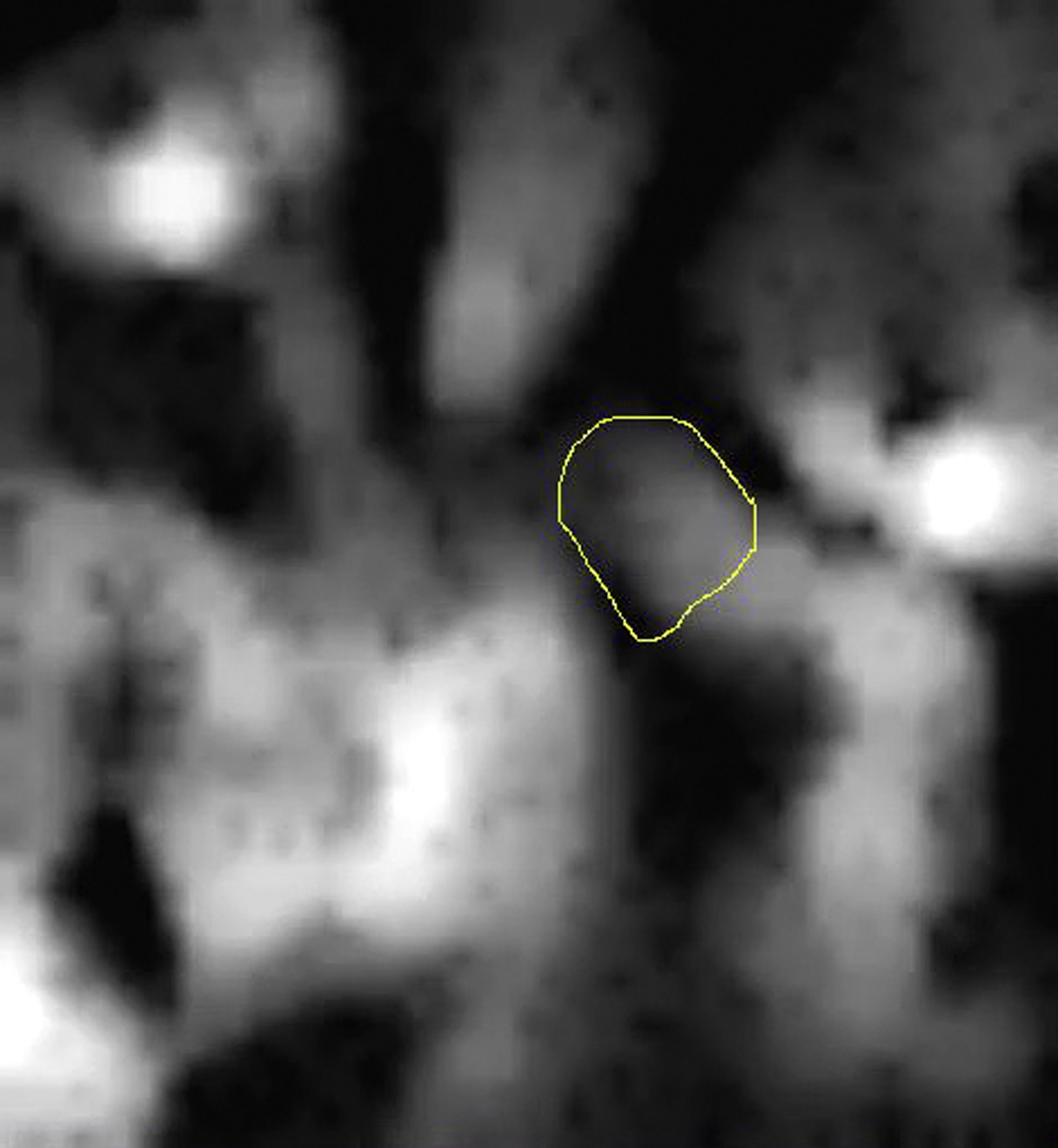

Supplement: Movie S1. Dynamics of Hub Function [file mmc2.jpg]

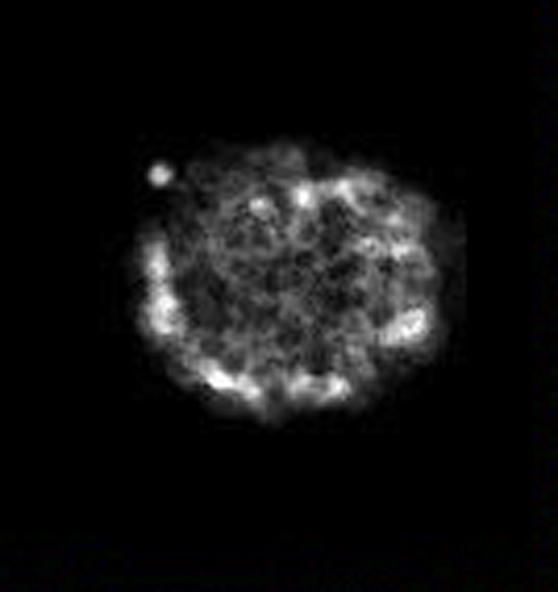

Supplement: Movie S2. Optogenetic Silencing of β Cell Function [file mmc3.jpg]

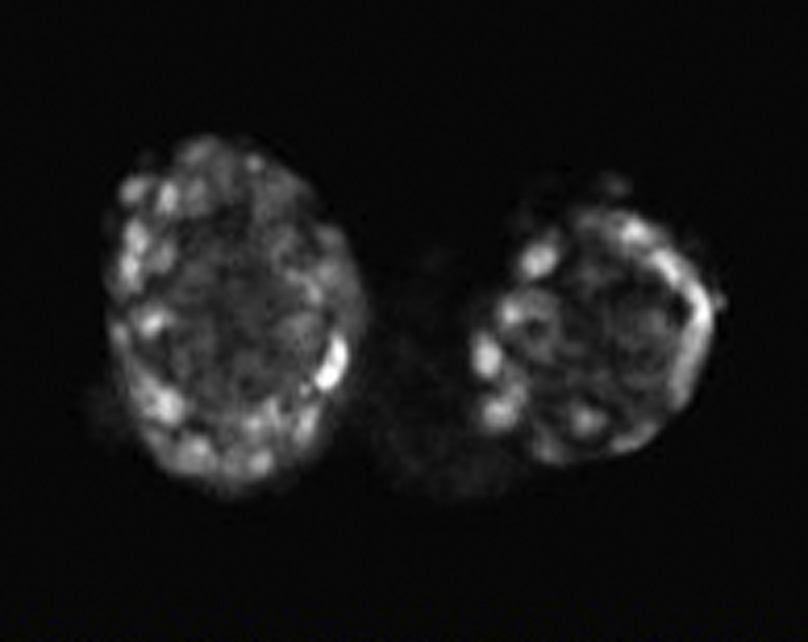

Supplement: Movie S3. Optogenetic Targeting of β Cells in a Single Islet [file mmc4.jpg]

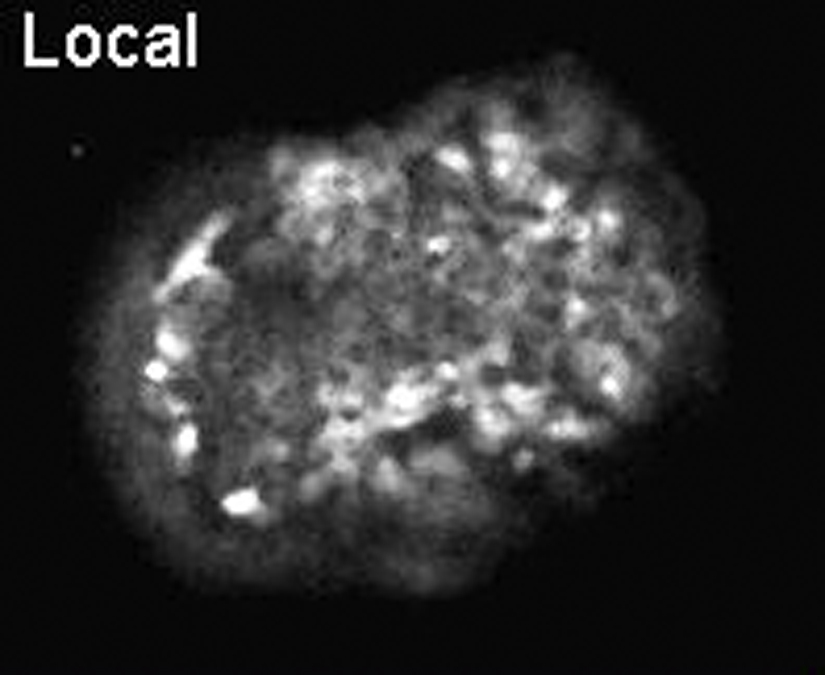

Supplement: Movie S4. Optogenetic Targeting of β Cells in an Islet Subregion [file mmc5.jpg]

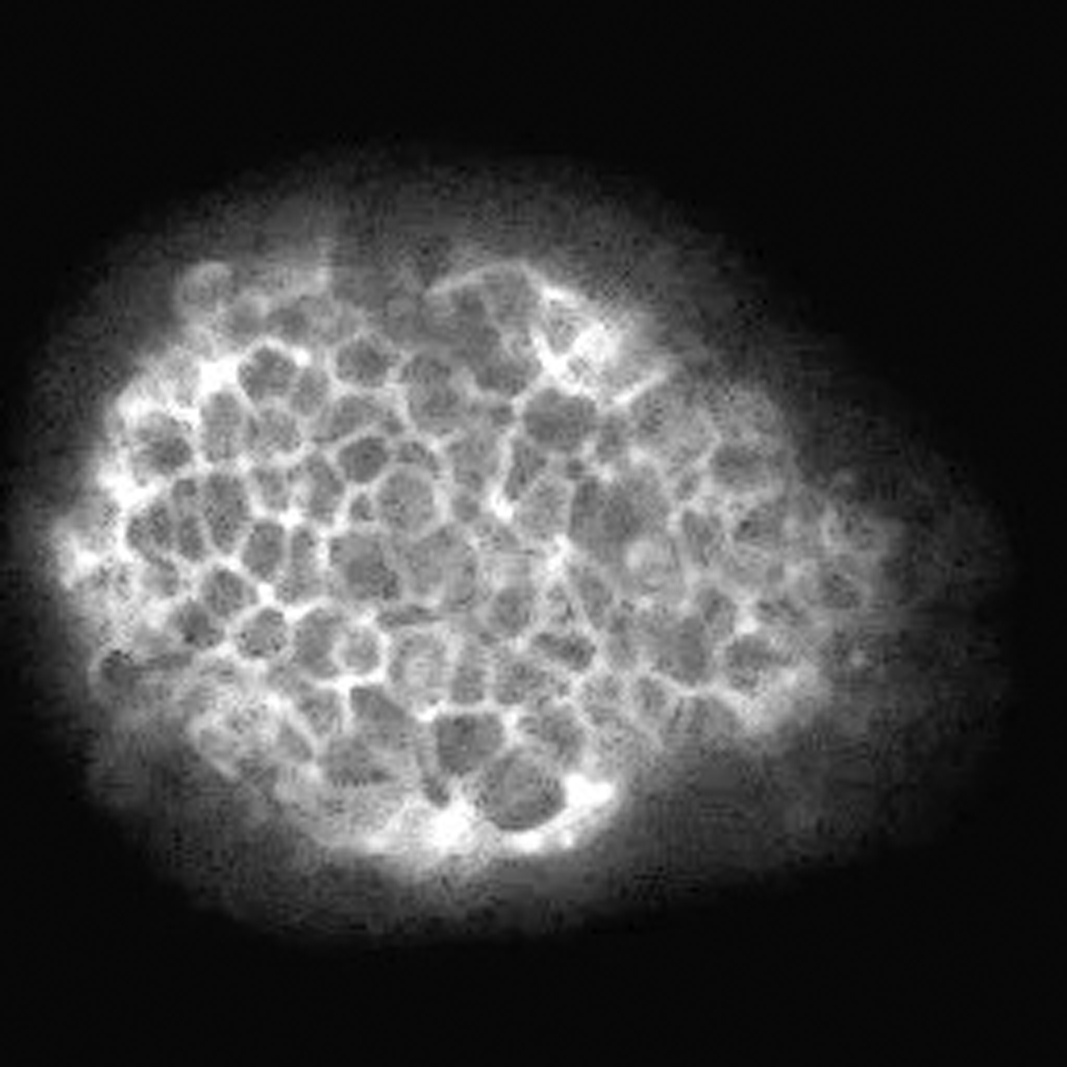

Supplement: Movie S5. β Cell Population Responses before Hub Silencing [file mmc6.jpg]

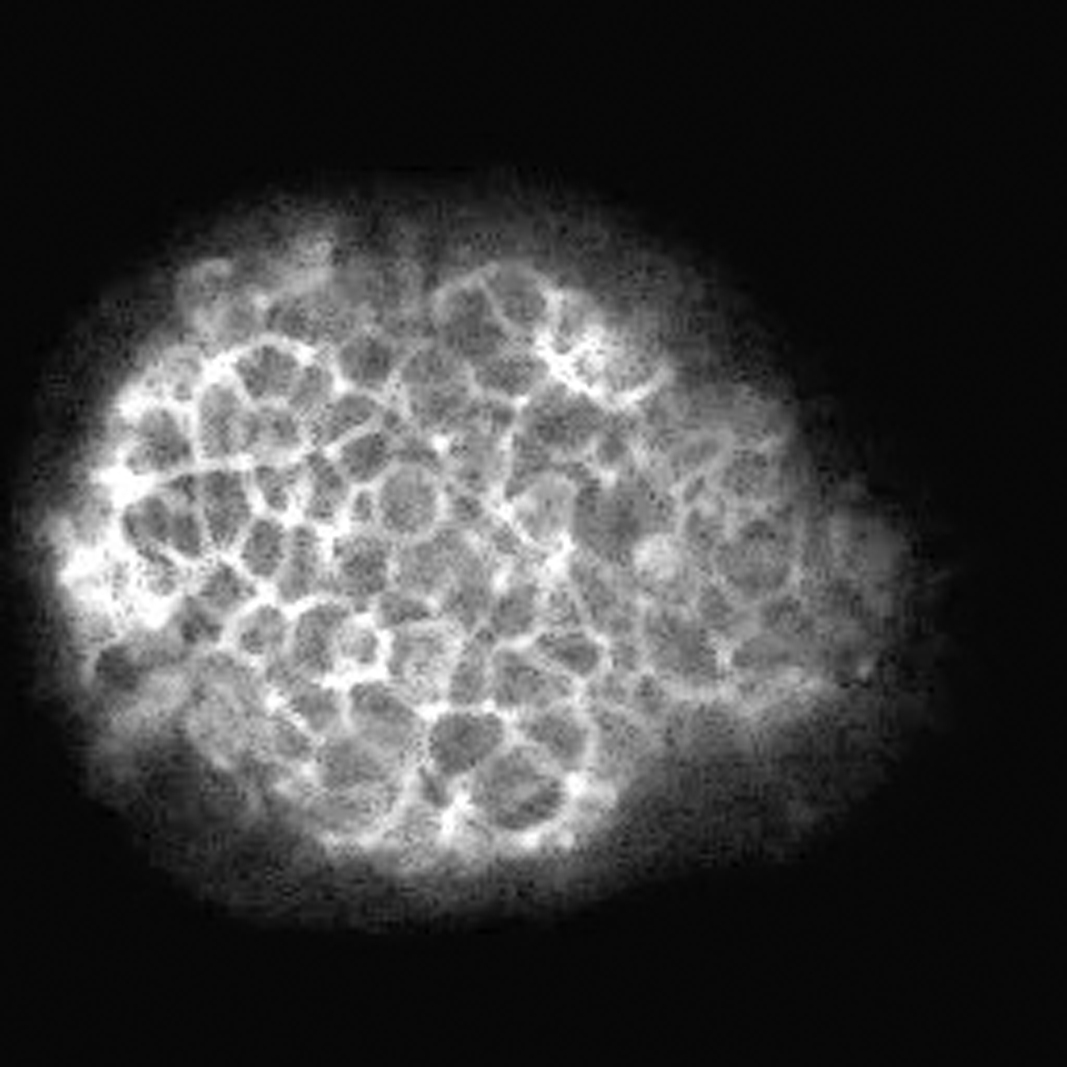

Supplement: Movie S6. β Cell Population Responses during Hub Silencing [file mmc7.jpg]
