# Supplementary material for: Beta Cell Hubs Dictate Pancreatic Islet Responses to Glucose
Source: Cell Metab. 2016 Sep 13;24(3):389–401. doi: 10.1016/j.cmet.2016.06.020 (PMC5031557; doi:10.1016/j.cmet.2016.06.020)
Supplement: Document S2. Article plus Supplemental Information [file mmc8.pdf]

Cell Metabolism

Article

Beta Cell Hubs Dictate Pancreatic Islet Responses to Glucose

Graphical Abstract

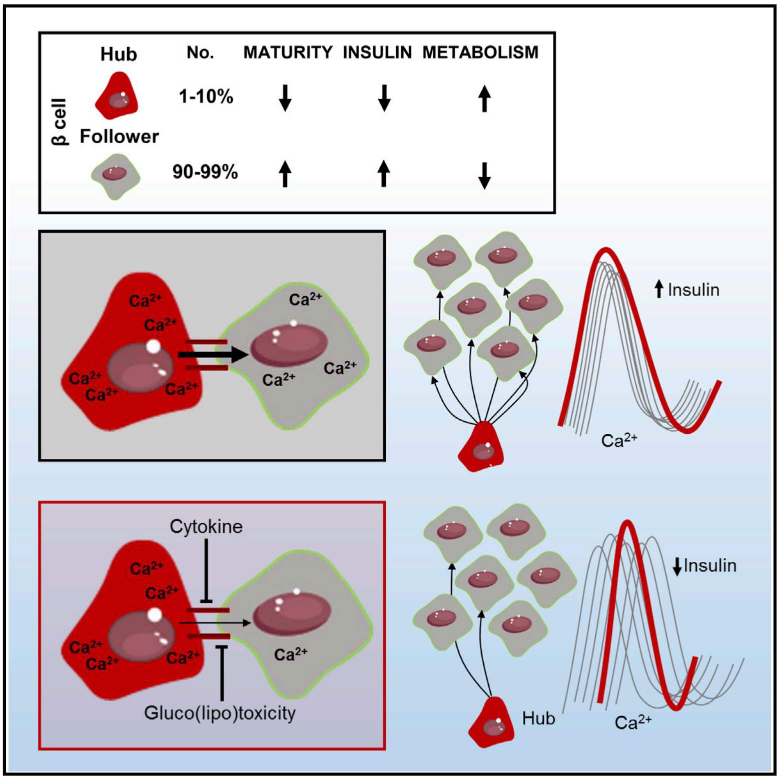

Authors

Natalie R. Johnston, Ryan K. Mitchell, Elizabeth Haythorne, ..., Dirk Trauner, Guy A. Rutter, David J. Hodson

Correspondence

g.rutter@imperial.ac.uk (G.A.R.), d.hodson@bham.ac.uk (D.J.H.)

In Brief

Combining optogenetics and photopharmacology, Johnston et al. show that a few (1%–10%) β cells exert disproportionate control over islet responses to glucose. These specialized cells, called hubs, are transcriptionally immature and highly metabolic. Their failure during type 2 diabetes mellitus may lead to reduced insulin secretion and impaired glucose homeostasis.

Highlights

- Optogenetic targeting reveals a pacemaker-like β cell subpopulation
- These cells, termed hubs, are required for normal insulin release
- Hubs are highly metabolic and transcriptionally immature
- Hubs are targeted by a diabetic milieu to induce islet failure

Johnston et al., 2016, Cell Metabolism *24*, 389–401
September 13, 2016 © 2016 The Author(s). Published by Elsevier Inc.
http://dx.doi.org/10.1016/j.cmet.2016.06.020

Cell Metabolism
Article

CellPress

Beta Cell Hubs Dictate Pancreatic Islet Responses to Glucose

Natalie R. Johnston,[1] Ryan K. Mitchell,[1] Elizabeth Haythorne,[1] Maria Paiva Pessoa,[1] Francesca Semplici,[1] Jorge Ferrer,[2] Lorenzo Piemonti,[3] Piero Marchetti,[4] Marco Bugliani,[4] Domenico Bosco,[5] Ekaterine Berishvili,[5] Philip Duncanson,[6] Michael Watkinson,[6] Johannes Broichhagen,[7] Dirk Trauner,[7] Guy A. Rutter,[1,*] and David J. Hodson[1,8,9,10,*]

[1]Section of Cell Biology and Functional Genomics, Division of Diabetes, Endocrinology and Metabolism, Department of Medicine, Imperial College London, London W12 0NN, UK
[2]Beta Cell Genome Regulation Lab, Department of Medicine, Imperial College London, London W12 0NN, UK
[3]Diabetes Research Institute (HSR-DRI), San Raffaele Scientific Institute, Via Olgettina 60, 20132 Milan, Italy
[4]Department of Clinical and Experimental Medicine, Islet Cell Laboratory, University of Pisa, 56126 Pisa, Italy
[5]Cell Isolation and Transplantation Center, Department of Surgery, Geneva University Hospitals and University of Geneva, 1205 Geneva, Switzerland
[6]School of Biological and Chemical Sciences, Queen Mary University of London, Mile End Road, London E1 4NS, UK
[7]Department of Chemistry, Ludwig-Maximilians-Universität München, and Munich Center for Integrated Protein Science, Butenandtstrasse 5-13, 81377 München, Germany
[8]Institute of Metabolism and Systems Research (IMSR) and Centre of Membrane Proteins and Receptors (COMPARE), University of Birmingham, Birmingham B15 2TT, UK
[9]Centre for Endocrinology, Diabetes and Metabolism, Birmingham Health Partners, Birmingham B15 2TH, UK
[10]Lead Contact
*Correspondence: g.rutter@imperial.ac.uk (G.A.R.), d.hodson@bham.ac.uk (D.J.H.)
http://dx.doi.org/10.1016/j.cmet.2016.06.020

SUMMARY

The arrangement of β cells within islets of Langerhans is critical for insulin release through the generation of rhythmic activity. A privileged role for individual β cells in orchestrating these responses has long been suspected, but not directly demonstrated. We show here that the β cell population in situ is operationally heterogeneous. Mapping of islet functional architecture revealed the presence of hub cells with pacemaker properties, which remain stable over recording periods of 2 to 3 hr. Using a dual optogenetic/photopharmacological strategy, silencing of hubs abolished coordinated islet responses to glucose, whereas specific stimulation restored communication patterns. Hubs were metabolically adapted and targeted by both pro-inflammatory and glucolipotoxic insults to induce widespread β cell dysfunction. Thus, the islet is wired by hubs, whose failure may contribute to type 2 diabetes mellitus.

INTRODUCTION

The release of insulin from pancreatic β cells is necessary for proper glucose homeostasis in mammals. β cells respond to glucose with increased oxidative metabolism, elevations in cytosolic ATP/ADP ratio, and closure of ATP-sensitive K^+ (K_{ATP}) channels (Rutter et al., 2015). The consequent plasma membrane depolarization activates voltage-dependent Ca^{2+} channels (VDCCs), leading to Ca^{2+} influx and exocytosis of secretory granules (Rutter et al., 2015).

The 3D organization of β cells is also important for the normal regulation of insulin secretion. Thus, β cells throughout the islet microorgan display rhythmic activity patterns in the presence of high glucose (Benninger et al., 2008; Santos et al., 1991). A role for specialized β cells in orchestrating these dynamics has long been postulated, including the presence of putative "pacemakers" (Ammälä et al., 1991; Benninger et al., 2014; Squires et al., 2002). Indeed, isolated β cells possess discrete metabolic characteristics and secretory profiles (Katsuta et al., 2012; Kiekens et al., 1992; Salomon and Meda, 1986), and phase lags in the onset of electrical activity can be detected between distant islet regions (Benninger et al., 2008; Meda et al., 1984; Palti et al., 1996). More recent studies have revealed functional differences between hundreds of individual β cells monitored in situ in the intact islet (Hodson et al., 2013; Li et al., 2011; Stožer et al., 2013). Such heterogeneity may be relevant for type 2 diabetes pathogenesis, since specific insults might target single cells or defined islet regions to induce insulin secretory failure. However, whether particular subsets of cells drive the behavior of others has so far been difficult to prove empirically.

Over the past decade, optogenetics has allowed reversible control of neuronal activity with light (Zhang et al., 2007). In parallel, photopharmacology has harnessed the power of azobenzene photoresponsive units to produce exogenously applied compounds that turn ion channels and G protein-coupled receptors into endogenous photoswitches (Broichhagen et al., 2015). As both these approaches are applicable to electrically excitable endocrine tissue (Broichhagen et al., 2014; Reinbothe et al., 2014), they afford the unique opportunity to precisely manipulate cell activity with high spatiotemporal fidelity. Using all-optical interrogation of individual β cells in situ, we therefore set out to probe the topology that regulates population glucose responsiveness, with the aim of identifying the islet-resident pacemaker.

This is an open access article under the CC BY license (http://creativecommons.org/licenses/by/4.0/).

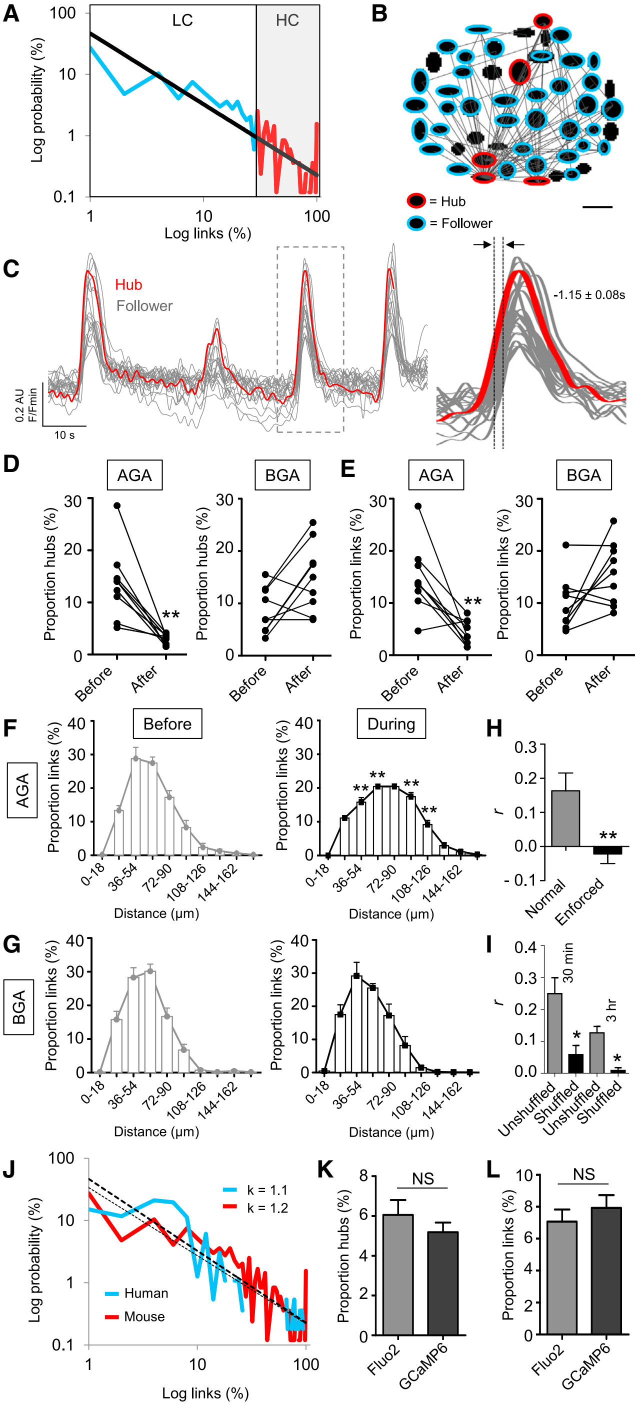

Figure 1. Functional Mapping of β Cell Population Dynamics

(A) At elevated glucose (11 mM), islets house a scale-free network where a few (<10%) β cells host the majority of correlated links ($p < 0.01$), as shown by the power law-fitted probability distribution (LC, low connectivity range; HC, high connectivity range) ($R^2 = 0.72$) (n = 12 recordings from five animals). A log-log scale is used to convert the power law into a linear relationship.

(B) Representative functional connectivity map displaying the x-y position of analyzed cells and their links (followers, blue; hubs, red; scale bar, 20 μm).

(C) Representative trace showing that hub (red) activity tends to precede and outlast that of follower cells (gray) (mean lag value calculated from n = 5 recordings from three animals).

(D) Treatment of islets with the gap junction blocker 18α-glycyrrhetinic acid (AGA; 20 μM) (left), but not its inactive analog glycyrrhizic acid (BGA; 20 μM) (right) reduces the proportion of hubs (n = 9 recordings from five animals) (before, islet in control buffer; after, same islet in the presence of either AGA or BGA).

(E) As for (D), but the proportion (%) of correlated links.

(F and G) Gap junction blockade increases the length between correlated links (n = 9 recordings from five animals).

(H) Wiring patterns are statistically stable upon re-recording after 30 min, as determined against the same islet but with enforced dissimilarity (n = 8 recordings from five animals).

(I) Wiring patterns are statistically stable upon re-recording after 30 min (Fluo2) and 3 hr (GCaMP6), as compared to the randomly shuffled correlation matrix for each islet (n = 4–6 recordings from two to three animals).

(J) As for (A) but showing almost identical link-probability distributions in mouse and human islets, as shown by the exponent values (κ) for the fitted power laws (n = 8 recordings from three donors).

(K and L) Imaging using GCaMP6 and Fluo2 return similar hub and link proportions (n = 12 recordings from four to six animals).

Data are means ± SEM.*$p < 0.05$ and **$p < 0.01$. NS, non-significant. See also Figure S1 and Movie S1.

RESULTS

Hubs Are a Feature of β Cell Population Dynamics

To visualize the large-scale organization of β cell activity underlying calcium (Ca^{2+})-dependent exocytosis of insulin granules, intact mouse islets were subjected to high-speed (2–8 Hz) multicellular Ca^{2+} imaging (Hodson et al., 2012). This was combined with Monte Carlo-based correlation analyses in which repeated shuffling of Ca^{2+} events (>9,999 iterations) is used to determine whether cells are coordinated due to chance or not (i.e., contributing to the same insulin release event). Together, these approaches allow online mapping of the islet functional circuitry. Initial experiments confirmed that β cells form a scale-free network (Stožer et al., 2013), which supports the synchronous propagation of glucose (11 mM)-stimulated Ca^{2+} waves by efficiently connecting distant islet regions ($R^2 = 0.72$; Figure 1A). Scale-free networks are ubiquitous throughout biology, are identified by their power law link-probability distribution (Hodson et al., 2010), and adopt a hub and spoke formation where a few cells possess the majority of connections. Accordingly, a stereotypical feature of such topology in islets was the non-random appearance of rare super-connected hubs, whose firing activity tended to repetitively precede and outlast that of the remainder of the population (i.e., was pacemaker-like) (Figures 1B and 1C) (Movie S1).

Such islet architecture was dependent on information exchanges through gap junctions, since reversible blockade of connexin channels using 18α-glycyrrhetinic acid (AGA) (Farnsworth et al., 2014) reduced the number of hubs, decreased coordinated population activity, and increased signal propagation path length (Figures 1D–1G). This may reflect the inability to identify hubs due to loss of cell-cell entrainment, as well as re-routing of information over longer distances by the remaining hubs. Notably, no differences in the amplitude of Ca^{2+} rises were seen in control and AGA-treated tissue (Figures S1A–S1C), suggesting minimal impact upon VDCC activity. In all cases, parallel experiments were performed using glycyrrhizic acid (BGA), the inactive precursor of AGA that exerts similar non-specific effects (Desarménien et al., 2013). Results could

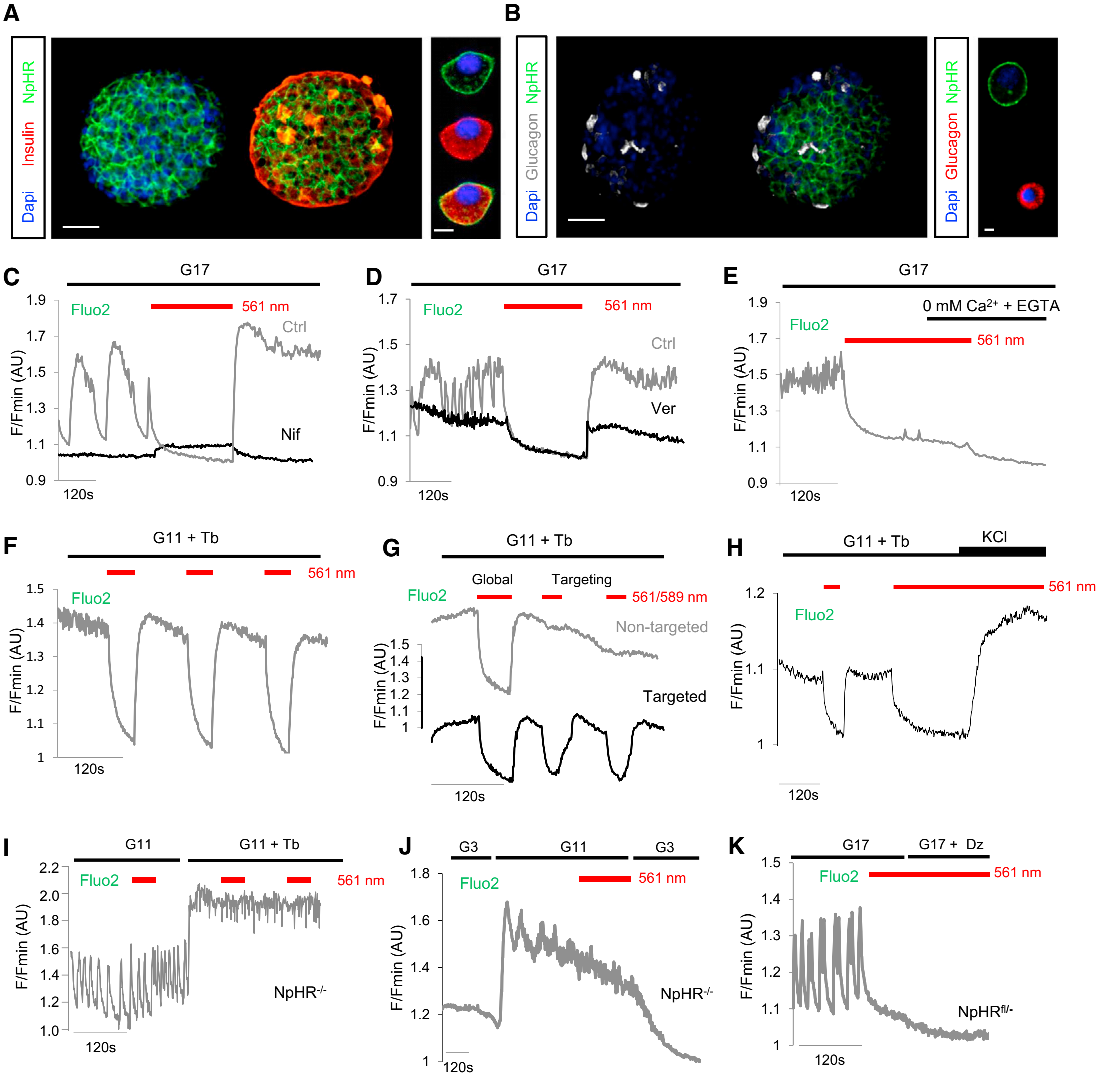

Figure 2. Reversible and Repeated Silencing of β Cell Ca^{2+}-Spiking Activity

(A) Immunostaining for insulin showing membrane-localized expression of eNpHR3.0-EYFP in β cells (Dapi, nuclei) (n = 3 preparations).

(B) As for (A), but immunostaining for glucagon showing absence of eNpHR3.0-EYFP in α cells (n = 3 preparations). Scale bar, 50 μm (or 10 μm for dissociated cells).

(C) Reversible silencing of β cell Ca^{2+} oscillations in eNpHR3.0-expressing islets in response to illumination with λ = 561 nm (n = 5 recordings). Treatment of islets with nifedipine 50 μM (Nif; black trace) abolishes the rebound in Ca^{2+} upon inactivation of eNpHR3.0 (n = 5 recordings) (traces are from different islets).

(D) As for (C), but in the presence of verapamil 10 μM (Ver; black trace) (n = 5 recordings) (traces are from different islets).

(E) Perifusion of islets with zero Ca^{2+} supplemented with EGTA was able to prevent recovery of $[Ca^{2+}]_i$ in islets following silencing (n = 5 recordings).

(F) β cell population Ca^{2+}-spiking activity can be repeatedly silenced following exposure to λ = 561 nm (n = 3 recordings).

(G) Global silencing (λ = 561 nm) induced a decrease in intracellular Ca^{2+} concentrations ($[Ca^{2+}]_i$) throughout the islet, whereas a diffraction-limited laser (λ = 589 nm) only silenced $[Ca^{2+}]_i$ in the targeted area (n = 3 recordings).

(H) Silencing can be overcome using the depolarizing agent KCl 30 mM to re-activate VDCC (n = 6 recordings).

(I and J) Wild-type islets (*NpHR*$^{-/-}$) do not respond to illumination with decreases in $[Ca^{2+}]_i$ (n = 5 recordings).

(K) Diazoxide (Dz) 100 μM is unable to further suppress $[Ca^{2+}]_i$ in eNpHR3.0-silenced islets (n = 5 recordings).

Where indicated, tolbutamide (Tb) 100 μM was added to maintain a stable plateau from which to better detect silencing. G17, glucose 17 mM; G11, glucose 11 mM; G3, glucose 3 mM. See also Movies S2, S3, and S4.

be replicated using mebeverine (Farnsworth et al., 2014) (Figure S1D), a gap junction inhibitor with no reported effects on VDCC or K_{ATP} channel activity, as well as *Gjd2* shRNA to specifically silence connexin-36 at the islet surface (Figures S1E–S1H).

Hubs Are Stable and Present across Species

To assess network topology stability, islets were recorded and then left on the microscope for between 30 min and 3 hr before re-recording. Network topology was stable both over time and in response to perturbation, as statistically assessed

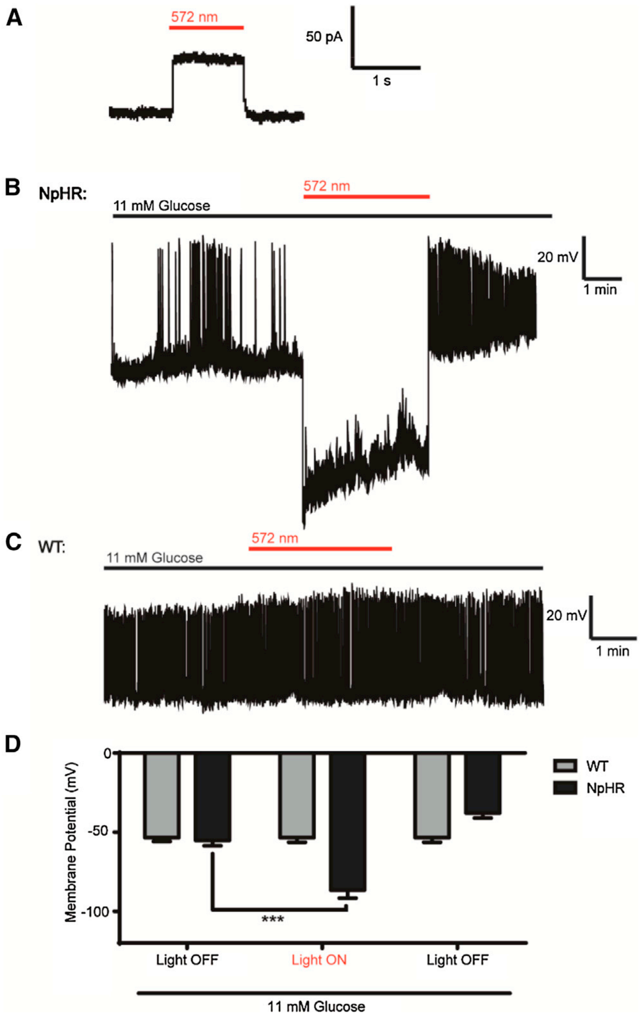

Figure 3. Yellow Light Hyperpolarizes eNpHR3.0-Expressing Pancreatic β Cells

(A) Voltage clamp (whole cell) recording of an eNpHR3.0-expressing β cell showing induction of photocurrents with yellow light (λ = 572 nm).

(B–D) Representative current clamp (perforated patch) recordings showing reversible membrane hyperpolarization with yellow light (λ = 572 nm) in an eNpHR3.0-expressing (NpHR), but not wild-type (WT), β cell. In all cases, n = 6–11 cells.

Data are means ± SEM. ***$p < 0.001$.

versus a third experiment subjected to either randomization (i.e., to re-distribute the wiring pattern) or enforced-dissimilarity (i.e., to form a different wiring pattern) (Figures 1H and 1I). Network indices were unaffected in the presence of either a specific glucagon receptor antagonist (Figures S1I and S1J) or a glucagon-neutralizing antibody (Figures S1K and S1L), suggesting that any glucagon present in vitro is unlikely to influence hub function. Hinting at a conserved role for hub architecture, islet functional topologies were similar in glucose-stimulated mouse and human islets, as shown by the similar link-probability distributions (i.e., both are fitted with a power law of near-identical exponent value) (Figure 1J). However, synchrony tended to be compartmentalized into subregions/clusters in human islets (Figure 1J), in line with the different structural arrangement of β versus α cells in this species (Bosco et al., 2010). β cell Ca^{2+} responses were not dependent on orientation toward the islet center or periphery (ΔY Fluo2 = 0.14 ± 0.01 versus 0.13 ± 0.004 AU, periphery versus center, respectively), and identical results were obtained using the geneticall encoded indicator GCaMP6 (Figures 1K and 1L), engineered to interfere less with intracellular Ca^{2+} levels.

A Strategy for All-Optical Interrogation of β Cell Function

To functionally dissect the role of hubs, an optogenetic strategy was developed and validated, enabling electrical silencing following *Ins1Cre*-directed expression of the light-gated chloride (Cl^-) pump halorhodopsin (eNpHR3.0) (Zhang et al., 2007) in β cells (Figures 2A and 2B). This approach allowed the reversible silencing of single β cell or population Ca^{2+}-spiking activity and extracellular Ca^{2+} influx following illumination (λ = 560–590 nm) (Figures 2C–2G) (Movies S2, S3, and S4). Application of the depolarizing agent potassium chloride was able to overcome silencing by restoring VDCC activity (Figure 2H). Of note, wild-type β cells were refractory to silencing (Figures 2I and 2J), and eNPHR3.0-expressing β cells under irradiation were not further hyperpolarized using diazoxide to force open K_{ATP} channels (Figure 2K). As measured using patch-clamp electrophysiology, illumination induced photocurrents (Figure 3A), leading to membrane hyperpolarization and electrical silencing only in eNPHR3.0-expressing β cells (Figures 3B–3D). Thus, specific and powerful optogenetic silencing could be achieved.

Animals harboring a single eNpHR3.0 allele unexpectedly demonstrated improved glucose tolerance compared to wild-type littermates, despite normal insulin sensitivity (Figures 4A–4F) and body weight/growth curves (Figures 4G and 4H). This was probably due to enhanced in vivo insulin secretion (Figure 4I), as β cell mass was apparently normal (Figure 4J). Activation of eNpHR3.0 on an Ins1Cre background also led to similar results, suggesting that alternation in insulin gene dosage in the context of the transgene was unlikely to be a contributing mechanism (Figures 4K and 4L). Pertinent to the in vitro studies here, however, isolated islets responded normally to glucose in terms of ionic fluxes and insulin release (Figures S2A–S2I), and eNpHR3.0 does not possess basal activity in the absence of light (Zhang et al., 2007) (also shown in Figure 3C).

Hubs Orchestrate β Cell Population Responses to Glucose

By performing analysis in real-time using islets maintained on the microscope stage, hubs could be identified and subsequently manipulated (Figures 5A–5C). Silencing of individual hubs using a pinpointing laser had catastrophic consequences for coordinated islet responses to high glucose (Figures 5D and 5E) (Movies S5 and S6), an effect reversed simply by ceasing illumination (Figures 5F and 5G). The strength of inhibition following targeting of individual hubs tended to be inversely associated with the number of these cells per islet before silencing (Figure S2J), suggesting that some redundancy is present in the system, most likely due to follower cells being controlled by more than one hub. By contrast, silencing of individual non-hub or follower cells did not significantly perturb islet dynamics (Figure 5H), demonstrating the specificity of the approach.

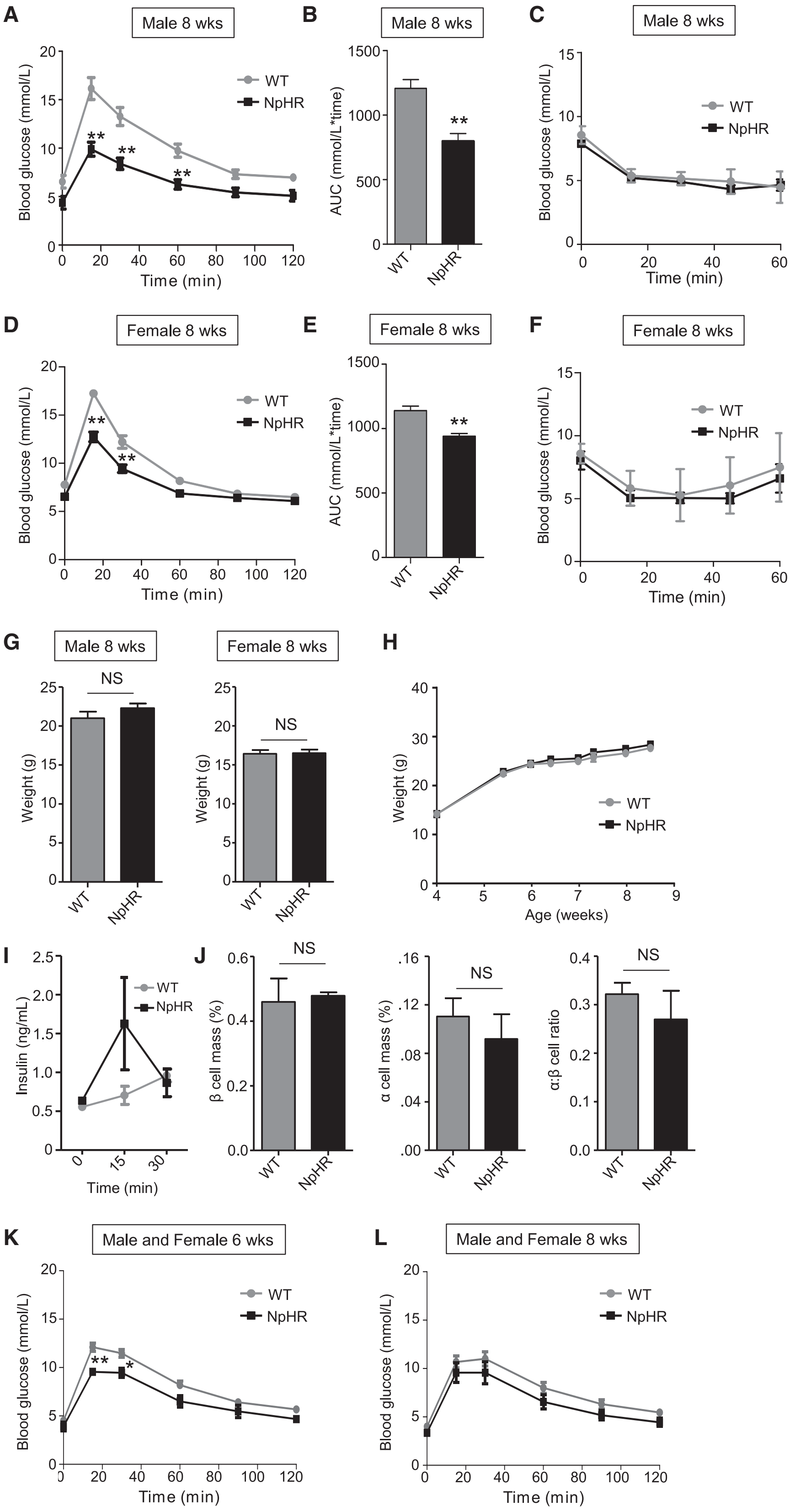

Figure 4. Glucose Homeostasis in eNpHR3.0 Mice

(A and B) Glucose tolerance is improved in male 8 week *Ins1Cre*$^{+/-}$;*eNpHR3.0-EYFP*$^{fl/-}$ (NpHR) animals (n = 7) compared to *Ins1Cre*$^{-/-}$; *eNpHR3.0-EYFP*$^{fl/-}$ (wild-type, WT) littermates (n = 7) (i.e., activation of Ins1Cre on an eNpHR3.0-EYFP background), as assessed using IPGTT.

(C) Insulin sensitivity is similar in male NpHR mice animals and WT littermates (n = 6–11), as determined using ITT.

(D and E) As for (A) and (B), but female 8 week mice (n = 7–9).

(F) As for (C), but female 8 week (n = 4).

(G and H) Fasting body weight and growth curves (non-fasted) are similar in WT and NpHR animals (n = 9–13).

(I) In vivo insulin release tended to be increased in NpHR compared to WT animals at 15 min post-IP glucose injection (n = 4).

(J) β cell mass, α cell mass, and α:β cell ratio are similar in WT and NpHR animals (n = 3).

(K and L) As for (A) and (B), but glucose tolerance in 6 and 8 week *Ins1Cre*$^{+/-}$;*eNpHR3.0-EYFP*$^{fl/-}$ (NpHR) (n = 3–4) compared to *Ins1Cre*$^{+/-}$; *eNpHR3.0-EYFP*$^{-/-}$ (wild-type, WT) animals (n = 8) (i.e., activation of eNpHR3.0-EYFP on an Ins1Cre-background).

Data are means ± SEM. **$p < 0.01$. NS, non-significant. See also Figure S2.

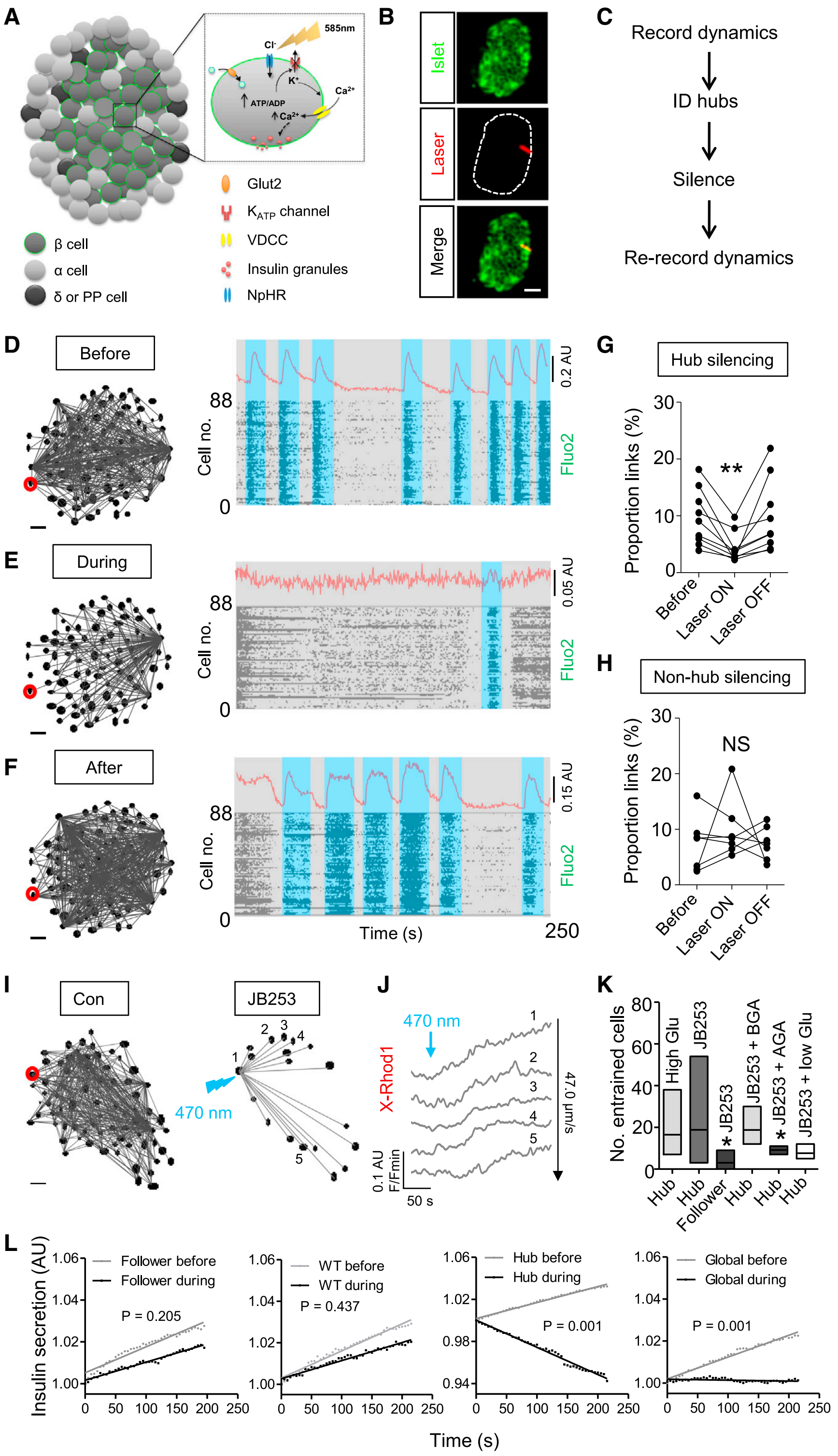

Figure 5. Real-Time Analysis and Targeting of β Cell Hubs

(A and B) Schematic showing the effects of eNpHR3.0 activation upon β cell Ca^{2+} signaling (A), and snapshot showing placement of a diffraction-limited laser spot over a discrete islet region (B) (scale bar, 25 μm; image cropped to display a single islet).

(C) Experimental flowchart for real-time manipulation of hub function.

(D–F) Representative functional connectivity map and activity plots at high glucose (11 mM), before (D), during (E) and after (F) optogenetic silencing (identified hub cell; red). A representative Ca^{2+} trace is displayed above.

(G and H) Summary data showing a reversible collapse in the proportion of correlated cell links following hub (G), but not follower (or non-hub) (H) silencing (n = 7–9 recordings from four animals).

(I–K) Representative cell-cell entrainment patterns (I) and representative Ca^{2+} rises in linked cells (J) following photopharmacological stimulation of an identified hub (red) at 3 mM glucose using JB253 (50 μM). Box and whiskers plot shows the range and mean number of hub- or follower-entrained cells under high (11 mM) glucose (High Glu) conditions, and following targeted stimulation using JB253 in the presence of control (3 mM glucose), BGA, AGA, and low (1 mM) glucose (low Glu) (K) (n = 4–7 recordings from three to four animals).

(L) Insulin secretion measured using JP-107 is unaffected following illumination of follower (or non-hub) cells or wild-type (WT) islets, but suppressed in response to hub or islet (global) shutdown (mean traces shown) (n = 8 islets from 4 animals).

Scale bars, 20 μm. Data are means ± SEM. *p < 0.05. NS, non-significant. See also Figures S2 and S3 and Movies S5 and S6.

Using a similar technique, hubs were first identified at high glucose, before inactivation using low glucose and stimulation with JB253, an exogenously applied K_{ATP} channel photoswitch based on glimepiride (Broichhagen et al., 2014). Following targeted illumination of JB253-treated islets, hub connectivity could be mimicked without activation of intervening cells, as determined by the presence of glucose- and gap-junction-dependent entrainment patterns in follower cells (conduction velocity = 47.0 ± 8.9 μm/s) (Figures 5I–5K). Such effects were unlikely to stem from diffusion of active JB253, since this molecule turns off within milliseconds in the dark (Broichhagen et al., 2014), and proximate cells remained unaffected by hub stimulation (Figures S3A–S3D).

Hubs Are Required for Insulin Secretion

We were unable to measure insulin secretion accurately from a single islet over the 5 min experimental period used here, since levels were below the detection sensitivity of current assays. Therefore, to link hub activity with hormone release, the cell-surface-attached fluorescent Zn^{2+} probe JP-107 (Pancholi et al., 2014) was instead employed as a surrogate to dynamically report Zn^{2+} co-released with insulin from cells at the islet surface, as previously reported with ZIMIR (Li et al., 2011). Using this

approach, silencing of follower cells or wild-type islets was without effect, as evidenced by a linear increase in fluorescence due to Zn^{2+} accumulation at the probe. By contrast, hub shutdown or global illumination lowered insulin/Zn^{2+} release to below the dissolution rate of the probe (i.e., Zn^{2+} binding is lower than Zn^{2+} removal) (Figure 5L).

While it was not technically possible to directly link hub activity with pulsatile insulin release, the acetylcholinomimetic carbachol (Zhang et al., 2008a) was able to accelerate β cell population activity (Figure S3E) without altering the proportion of links or hubs (Figures S3F and S3G). Moreover, rapid imaging performed over dozens of minutes—i.e, within the range of insulin pulses (Head et al., 2012)—revealed that hubs are also a feature of population behavior over longer periods (proportion hubs = 7.1% ± 1.3%; proportion links = 9.7% ± 2.0%). Since carbachol has been shown to phase-set activity between islets in vitro (Zhang et al., 2008a), parasympathetic neurons may plausibly target hubs in vivo to synchronize islet activity and generate insulin pulses.

Hubs Possess a Characteristic Metabolic Signature

We next sought to understand what makes a hub cell unique. Islet-wide Ca^{2+} signals were recorded before metabolic profiling of the hub population in the same islet using the mitochondrial potential dye tetramethylrhodamine ethyl ester (TMRE), which sequesters in active, hypeporlarized mitochondria. Following stimulation at high glucose, mitochondria in hubs became more hyperpolarized versus those in non-hubs (Figures 6A and 6B), suggesting increased proton pumping, ATP synthase activity, and ATP generation (Tarasov et al., 2012). While the duty cycle (i.e., proportion of time the cell spends "ON") was slightly increased in hubs compared to non-hubs (Figure 6C), other activity parameters including Ca^{2+}-spiking amplitude and frequency were broadly similar (Figures 6D–6F). Spatially, hubs and non-hubs were intermingled, with no clear preference for the islet center or periphery detected for either population based on polar coordinates (angle and distance taken from the islet center) (Figures 6G and 6H).

Hubs Display Features of both Mature and Immature β Cells

Using photoactivatable Tag-RFP (PA-TagRFP) to photopaint single hubs within islets using a 405 nm laser (Figures S4A and S4B), post hoc immunostaining against a variety of markers of β cell "identity" (Rutter et al., 2015) could be performed (Figure 6I) without adversely altering Ca^{2+} dynamics (Figure S4C). These studies revealed reduced insulin content, increased glucokinase (Gck) levels, lowered expression of pancreatic duodenum homeobox-1 (Pdx1), but normal levels of the mitochondrial import receptor subunit TOM20 homolog (Tomm20) in hubs versus the rest of the population (Figures 6I and 6J) (Figure S5). The transcription factor NK6 homeobox 1 (Nkx6.1), recently shown to be required for insulin biosynthesis and β cell proliferation (Taylor et al., 2013), was almost absent from hubs (Figures 6I and 6J). Suggesting that hubs are unlikely to represent a multihormonal (e.g., Glu+, Ins+) population, no co-localization with glucagon was detected (Figure 6K). Likewise, neurogenin 3 (Ngn3), a β cell precursor marker, was undetectable at the protein level in the adult islet, implying that hubs are unlikely to be trapped in a progenitor state (Figure 6K). Inspection of oversampled and deconvolved superresolution confocal images revealed no differences in mitochondrial distribution/shape or endoplasmic reticulum content in hubs (Figures 6L–6P), although expression of the sarco(endo)plasmic reticulum Ca^{2+}/ATPase, SERCA2, was markedly reduced (Figures 6O and 6P).

Suggesting a hyposecretory (or degranulated) nature, insulin granule numbers were lower in hubs versus non-hubs, despite a similar distribution (Figures 6Q and 6R). Furthermore, the area of individual hub cells was comparable to the rest of the population (range = 122–381 μm^2 and 194–355 μm^2, non-hubs versus hubs, respectively), and their shape appeared to be normal. Consequently, hubs constitute a metabolically adapted, repurposed subpopulation of β cell that displays features of immature cells.

Hubs Are Targeted by Diabetic Milieu

Lastly, the robustness of hubs was determined by challenging islets with cytokine cocktails (IL-1β/IL-6 or IL-1β/TNF-α) to recreate the pro-inflammatory milieu thought to be associated with diabetes (O'Neill et al., 2013). Acutely, the application of cytokines led to a large ramp-up in Ca^{2+} spiking activity in the presence of high glucose (Figure S6). However, after only 2 hr incubation, a collapse in hub cell number was apparent (Figures 7A and 7B), and this could be viewed in real-time by recording the same islet left in situ before and during exposure to cytokine (IL-1β/IL-6) (Figures 7C and 7D). The cytokine-induced disruption to hub cell function was further evidenced by a reduction in the number of cells occupying the upper or "high connectivity" region of the link-probability distribution (Figures 7E and 7F), as shown by a decrease in the exponent value of the power law fit. This resulted in a dramatic decline in correlated β cell population function (Figure 7G) due to the presence of fewer and less well-connected hubs. The actions of cytokines were not explained by effects on cell viability, as assessed using indices of necrosis (Figures 7H and 7I) and apoptosis (Figure 7J). However, 2 hr cytokine exposure decreased mRNA levels of the major islet gap junction isoform connexin-36 (*Gjd2*) 3-fold (Figures 7K and 7L), and this was already associated with a substantial reduction in gap junction plaque number (Figure 7M), in line with that recently reported using a similar paradigm (Farnsworth et al., 2015). Likewise, preferential hub failure was detected in both rodent and human tissue in response to gluco(lipo)toxic insults (Figures 7N and 7O).

DISCUSSION

β cells are a phenotypically diverse population, presenting a mosaic of metabolic and electrical activity patterns (Pralong et al., 1990), which is mirrored at the level of insulin secretory capacity (Katsuta et al., 2012; Kiekens et al., 1992; Li et al., 2011; Salomon and Meda, 1986). When viewed as a population, β cells are often termed a functional syncytium, although a role for cell heterogeneity in generating multicellular dynamics has been invoked repeatedly (Benninger and Piston, 2014; Stožer et al., 2013). Indeed, it has been shown that a subset (∼10%–15%) of β cells may exert a disproportionate influence over islet dynamics (Hraha et al., 2014). By combining large-scale functional cell mapping with optogenetics and photopharmacology, we

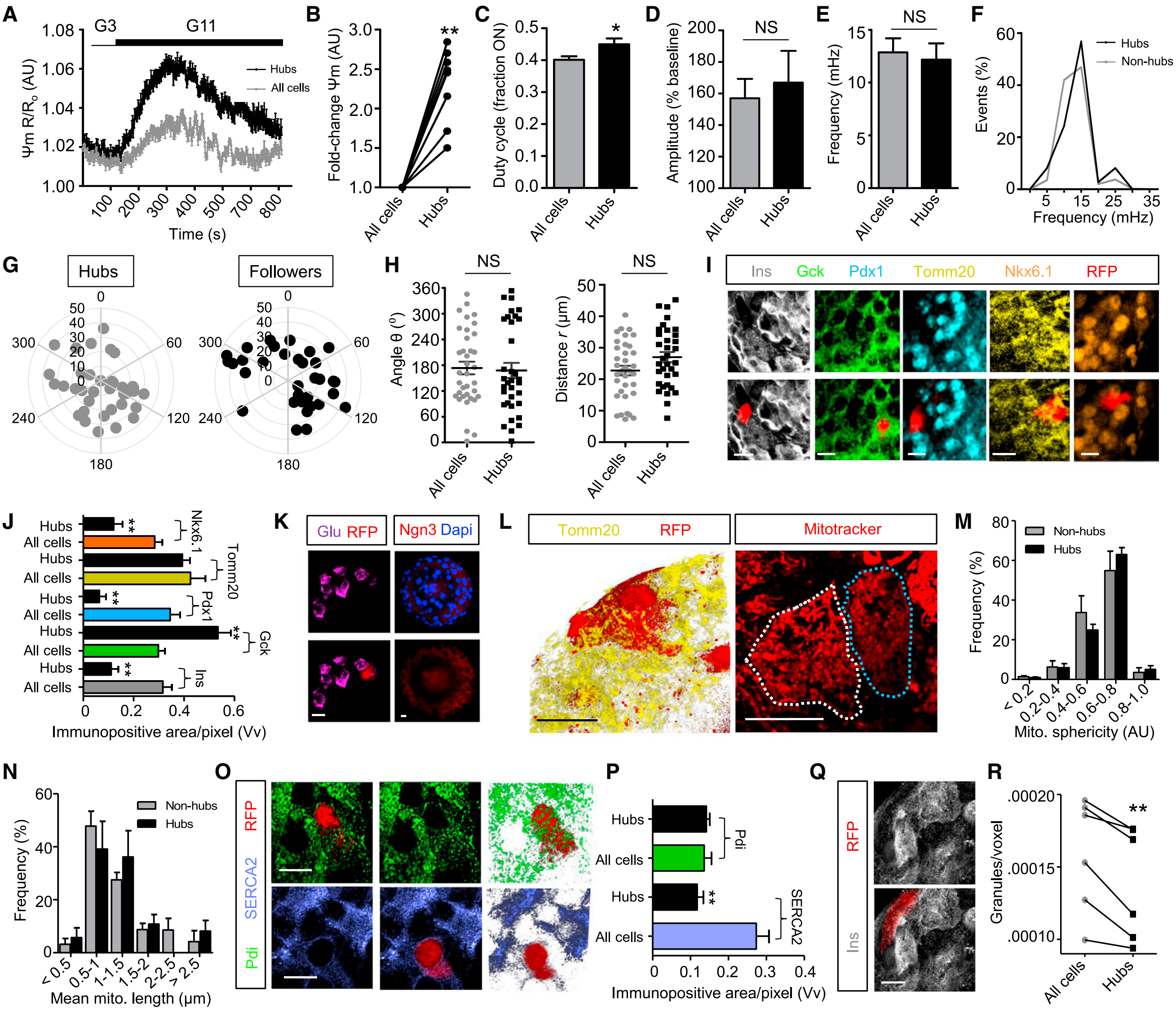

Figure 6. Phenotypic Profiling of Hub Cell Function

(A and B) Hubs display elevated mitochondrial potential (Ψm) compared to the rest of the population, as measured using TMRE to label active mitochondria (n = 9 recordings from three animals) (G3, glucose 3 mM; G11, glucose 11 mM) (fold-change is normalized to all cells).

(C–F) Duty cycle (i.e., fraction spent "ON") (C) and Ca^{2+} oscillation amplitude (D) and frequency ([E], [F]) are similar in hubs and followers (n = 8 recordings from four animals).

(G and H) Polar coordinates showing that hub distribution is not spatially biased versus followers (angle θ and distance r from the islet center 0,0 are shown in the bar graphs).

(I and J) PA-TagRFP-identified hubs (red; RFP) express less insulin (Ins), less Pdx1, less Nkx6.1, more Gck, and normal Tomm20 compared to the rest of the population (n = 5–9 hubs from three to four animals).

(K) Hubs were not immunopositive for glucagon (Glu), and Ngn3 expression was largely undetectable in the adult islet.

(L–N) High resolution Z projections of Tomm20- and MitoTracker-stained islets (L) reveal normal mitochondrial sphericity (M) and length (N) (white-dashed line, hub; blue-dashed line, non-hub) (3D render shown for Tomm20 and Z projection for MitoTracker) (n = 6 hubs from three animals).

(O and P) As for (L)–(N), but staining for Pdi and SERCA2 showing normal endoplasmic reticulum abundance and lowered Ca^{2+}-ATPase content in hubs (Z projection, left; 3D render, right) (n = 4 to 5 hubs from three animals).

(Q and R) High-resolution snapshot of insulin staining (Q) showing a reduction in granule content in hubs (red) (R) (n = 6 hubs from three animals). Scale bars, 12.5 μm. Data are means ± SEM. *p < 0.05 and **p < 0.01. NS, non-significant. See also Figures S4 and S5.

provide here a revised blueprint for islet function whereby a few pioneer hubs with reduced β cell identity dictate emergent population behavior in response to glucose. Importantly, hub topologies are a feature of dynamical systems, including cell networks in the brain and pituitary (Bonifazi et al., 2009; Hodson et al., 2012), since they are functionally robust at a low wiring cost (Bullmore and Sporns, 2009) (i.e., the chances of randomly hitting a hub are low). However, should a hub be specifically targeted,

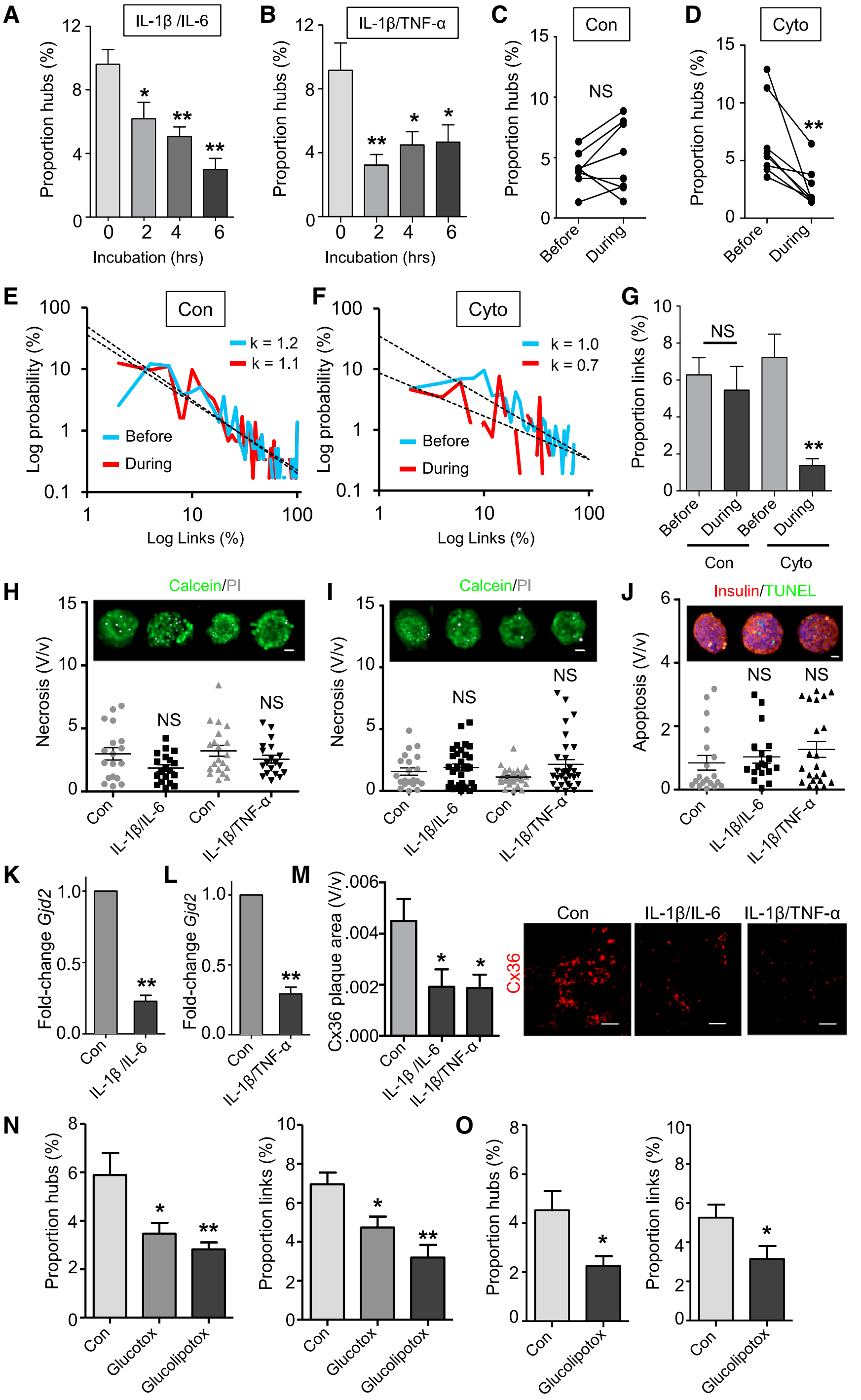

Figure 7. Disruption of Hubs by Pro-Inflammatory and Glucolipotoxic Insults

(A and B) IL-1β/IL-6 and IL-1β/TNF-α reduce hub number after 2 hr (n = 6 islets from three animals).
(C and D) Cytokine (Cyto; IL-1β/IL-6) decreases hub number in real-time (n = 8 from four animals).
(E and F) Cytokine alters the distribution of correlated links and power law scaling exponent (*k*) value, indicating a decreased number of cells in the high connectivity (i.e., hub) range (n = 8 from four animals) (R^2 = 0.38–0.74). The power law was log-log transformed to a linear relationship to better demonstrate the distribution.
(G) Cytokine (IL-1β/IL-6) exposure dramatically reduces the proportion of correlated links.
(H to I) Application of IL-1β/IL-6 or IL-1β/TNF-α for 2 hr (H) or 4 hr (I) is not cytotoxic (n = 21 islets per condition from 6 animals) (scale bar, 25 μm).
(J) 2 hr application of IL-1β/IL-6 or IL-1β/TNF-α does not induce apoptosis (n = 18–20 islets from five animals).
(K and L) IL-1β/IL-6 and IL-1β/TNF-α decrease connexin-36 (*Gjd2*) mRNA levels (n = 10 animals).
(M) IL-1β/IL-6 and IL-1β/TNF-α reduce the number of immunostained gap junction (connexin-36; Cx36) plaques (n = 9–12 islets from six animals) (scale bar, 12.5 μm).
(N) Glucotoxicity (Glucotox) and glucolipotoxicity (Glucolipotox) reduce the proportion of hubs and correlated links in mouse islets (n = 6 animals).
(O) As for (N), but showing effects of glucolipotoxicity-alone on human islets (n = 5 donors).
Control, Con (buffer-alone). Data are means ± SEM. *p < 0.05; **p < 0.01. NS, non-significant.
See also Figure S6.

with optogenetics. First, a diffraction-limited two-photon laser spot (i.e., ~500 nm) is insufficient to reliably activate optogenes, and the long excited state halftime can quickly saturate the rhodopsin (Rickgauer and Tank, 2009). Second, commercial lasers are unable to deliver the >1,100 nm excitation required for eNpHR3.0 activation without an optical-parametric oscillator (Andresen et al., 2009). By contrast, a single-photon diffraction-limited laser spot (~500 nm) of known absorbance cross-spectrum can be introduced to the surface of the sample, with minimal aberration and steep power drop-off as a function of 1/distance^2. Demonstrating the high degree of localization of the effective beam, we were clearly able to photopaint single cells within an islet and did not see any population silencing when a follower cell was targeted.

the effects on cell population function are far reaching, as observed in the islet during exposure to cytokine or glucolipotoxicity.

The present study used a single-photon-based confocal system to control the activity of individual hubs or followers within isolated islets. While two photon approaches in theory increase the accuracy of cell targeting by restricting the beam to within a few microns of the focal point, there are drawbacks when used

Using patch-clamp recordings of dissociated β cells, eNpHR3.0 activation hyperpolarized membrane potential by −60 mV, in line with previous reports (Mattis et al., 2012). While photocurrent size may be underestimated due to the presence of an electrochemical gradient, it should be noted that halorhodopsin derives

energy from photons rather than the ion gradient itself (Pfisterer et al., 2009), and the photocycle is unaffected even in the presence of high Cl^- concentration (Váró et al., 1995). In any case, it is unlikely that hyperpolarizing spread throughout the islet per se could account for these observations, since (1) only 30% of voltage spreads to an immediately coupled cell and an 86 mV depolarizing step is required for activation via gap junctions (Zhang et al., 2008b); and (2) stimulation of follower cells was without effect. We prefer an explanation whereby large changes in conductance attributable to the hub cell or its very close neighbors are removed through eNpHR3.0-mediated silencing, leading to impaired propagation of Ca^{2+} waves (Benninger and Piston, 2014; Benninger et al., 2008; Zhang et al., 2008b). Although membrane potential was slightly more depolarized following cessation of illumination, this is also seen in neurons (Mattis et al., 2012) and may reflect the reversal potential of Cl^-. We did not notice significant effects on hub indices during the Ca^{2+} imaging studies here due to use of a 5–10 min "rest" period to allow Cl^- re-equilibration.

Experiments in which hub cells were stimulated revealed that hubs and followers are unlikely to form local syncytia. While the exact mechanisms for antipodal signal propagation are difficult to determine precisely, a role for 3D chains of electrically coupled cells is plausible, given that entrainment was markedly blunted by both gap junction blockade and perfusion with 1 mM glucose. Other communication possibilities include autonomic neurons, which possess >100 μm axonal arborizations in pancreatic slices (Rodriguez-Diaz et al., 2012), and cilia, which provide a restricted signaling corridor due to their presence in only ~25% of β cells (Gerdes et al., 2014). Along these lines, the effect of hub silencing on islet function was surprisingly strong, given the relatively mild phenotype of animals deleted for the gap junction protein connexin36 (Ravier et al., 2005). However, considerable redundancy exists in the latter model with connexin-30.2 (Cx30.2) and ephrins providing alternative signaling routes (Farnsworth and Benninger, 2014; Konstantinova et al., 2007).

An intriguing possibility is that hubs are related to the previously described Pdx1+, Ins(low) β cell subpopulation (Szabat et al., 2009), albeit distinct in their low levels of both markers. Impaired identity, while conceivably restraining stimulus-induced secretion, may also limit GK-induced proliferation (Porat et al., 2011; Stolovich-Rain et al., 2015) to maintain the role of these cells as specialized pacemakers. Indeed, high levels of GK expression may sensitize hubs cells to increases in glucose concentration, allowing these cells to respond earlier and more robustly than their neighbors. By contrast, the failure of hubs when faced with a gluco(lipo)toxic/pro-inflammatory milieu indicates that these cells are metabolically fragile. This vulnerability might reflect high Gck/*Gck* (Roma et al., 2015) expression coupled to low Pdx1 and SERCA2 levels (Fonseca et al., 2011; Fujimoto et al., 2009), which ultimately lead to ER stress and cell dysfunction.

We acknowledge that the hub protein characterization performed here constitutes a biased screen, but it nonetheless provides a strong foundation for understanding the biology of these unusual cells. In the future, unbiased multiplex approaches, including massive parallel sequencing (RNASeq) and CyTOF (single cell mass cytometry) (Proserpio and Lonnberg, 2015), will help define the hub signature. Although attempts were made to obtain dissociated cells/cytoplasm for these purposes, PA-TagRFP fluorescence disappeared following dissociation of islets, possibly reflecting either the fragility of these cells, or the fluorophore itself. Similar problems were encountered with electron microscopy, where available antibodies cannot differentiate between activated and non-activated PA-TagRFP.

The recording approaches used to monitor hubs were technically constrained to 2 to 3 hr. Indeed, such experiments necessitate leaving the islets in situ on the microscope, since the same field of view must be maintained for analysis purposes. Thus, it cannot be excluded that hubs may represent a transitory subpopulation that drifts over dozens of hours in line with transcriptional/translational processes. Indeed, modeling studies predict that "pacemakers" arise from the most excitable β cell, which is assumed to shift due to a random distribution of excitability as K_{ATP} channel expression levels vary (Benninger et al., 2014). However, the possibility that such cells may arise during development could not be excluded (Benninger et al., 2014), and studies in FACS-purified GFP-labeled β cells suggest the presence of distinct transcriptional pools, with the proportions remaining similar between animals and days (Katsuta et al., 2012). Moreover, to the best of our knowledge, there is no evidence that K_{ATP} channel levels change over time, though the presence of a substantial proportion of channel subunits on internal membranes (Varadi et al., 2006) may complicate such measures.

Lastly, it should be noted that experiments in isolated islets may not necessarily reflect the situation in vivo, where blood flow direction (β cell → α cell) (Nyman et al., 2008) and molecule access dynamics (Michau et al., 2015) may all affect the role of hubs in dictating population dynamics and insulin secretion. This possibility might be tested in the future using in vivo imaging approaches (Nyman et al., 2008; Speier et al., 2008).

In summary, the present findings provide new insights into the regulation of islet function by individual β cells and the mechanisms that likely target and impair this during type 2 diabetes pathogenesis and treatment. More generally, the paradigm developed here to study the roles of individual cells within the functioning islet may be broadly applicable to other tissues or organisms.

EXPERIMENTAL PROCEDURES

Animals, Glucose/Insulin Tolerance Testing and Insulin Measures

β-cell-specific expression of halorhodopsin was achieved by crossing the Ins1Cre deletor strain (Thorens et al., 2015) with animals engineered to express eNpHR3.0-EYFP following excision of a *loxP*-flanked STOP cassette (B6;129S-Gt(ROSA)26Sortm39(CAG-hop/EYFP)Hze/J). *Ins1Cre*$^{+/-}$ and *Ins1Cre*$^{-/-}$ littermates (i.e., derived from an *Ins1Cre*$^{-/-}$ × *Ins1Cre*$^{+/-}$ breeding pair) display similar glucose tolerance and growth curves (Thorens et al., 2015), as well as insulin tolerance, in vivo and in vitro insulin secretion, and β cell mass (Figure S7). For detailed information, see Supplemental Experimental Procedures.

Islet Isolation

Islets were isolated using collagenase digestion and cultured for 24–72 hr in RPMI medium supplemented with 10% fetal calf serum, 100 U/ml penicillin, and 100 μg/ml streptomycin.

Human Islet Culture

Human islets were obtained from isolation centers in Italy and Switzerland, with necessary local and national ethical permissions, including consent from the next of kin. Islets were cultured in RPMI supplemented with 10% fetal calf serum, 100 U/ml penicillin, 100 μg/ml streptomycin, and 0.25 mg/ml fungizone, supplemented with 5.5 mM *D*-glucose. Ethical approval was granted by the National Research Ethics Committee London (Fulham), REC #07/H0711/114.

Calcium and Mitochondrial Potential Imaging

Multicellular Ca^{2+} and mitochondrial potential imaging was performed as detailed in Hodson et al. (2013). Mitochondrial potential was monitored using TMRE. For detailed information, see Supplemental Experimental Procedures.

Electrophysiology

Pancreatic islets were dissociated into single β cells and plated onto glass coverslips. Electrophysiological recordings were performed in either perforated patch-clamp or whole-cell configuration using an EPC9 patch-clamp amplifier controlled by Pulse acquisition software (HEKA). For detailed information, see Supplemental Experimental Procedures.

Dynamic Insulin Secretion Measures

Zinc (Zn^{2+}) co-released from insulin-containing granules was measured as a proxy for insulin secretion using the chemical probe JP-107 (300 μM), as described in Pancholi et al. (2014). For detailed information, see Supplemental Experimental Procedures.

Generation of Adenoviral PA-TagRFP and Photopainting

cDNA encoding the photoactivatable fluorescent protein PA-TagRFP (Subach et al., 2010) was cloned into pShuttleCMV *via Xho 1* and *Xba I* sites before recombination with pAdEasy1 and virus production as described in Luo et al. (2007). Islets were incubated for 48 hr with adenovirus harboring PA-TagRFP at a MOI = 100. For detailed information, see Supplemental Experimental Procedures.

shRNA-Silencing of Connexin-36

For detailed information, see Supplemental Experimental Procedures.

Immunohistochemistry

Islets were fixed at 4°C overnight in paraformaldehyde (4%, wt/vol) before permeabilization (PBS + Triton 0.1%) and application of primary and secondary antibody for 24–48 hr at 4°C. Connexin-36 staining was performed as above, but following 10 min fixation in ice-cold acetone. For detailed information, see Supplemental Experimental Procedures.

β and α Cell Mass

For detailed information, see Supplemental Experimental Procedures.

Necrosis and Apoptosis Assays

For detailed information, see Supplemental Experimental Procedures.

Real-Time PCR

Relative mRNA abundance was determined on an Applied Biosystems ABI 7500 Fast Real-Time PCR System using SYBR Green reagents and primers against connexin 36 (*Gjd2*) (GATTGGGAGGATCCTGTTGAC and AGG GCTAGGAAGACAGTAGAG). Gene expression was normalized to β-actin (CGAGTCGCGTCCACCC and CATCCATGGCGAACTGGTG) and fold-change in mRNA expression compared to control was calculated using the $2^{-\Delta\Delta CT}$.

Correlation, Similarity Analyses and Polar Coordinates

Individual EYFP-expressing β cells were identified using an ROI to produce a mask overlay of the imaged population. Correlation analyses were then performed in MATLAB on Hilbert-Huang transformed Ca^{2+} signals using binarization and matrix analyses, and statistical significance assigned using non-deterministic (Monte-Carlo) methods, as described (Hodson et al., 2010, 2012). For detailed information, see Supplemental Experimental Procedures.

Measurements of Insulin Secretion from Isolated Islets

For detailed information, see Supplemental Experimental Procedures.

Cytokines and Glucolipotoxicity

Interleukin 1 β (IL-1β), interleukin 6 (IL-6), and tumor necrosis factor α (TNF-α) (all from R&D Biosystems) were stored as stock solutions at −20°C and used at 20 pg/ml, 40 pg/ml, and 20 pg/ml, respectively (Maedler et al., 2004; O'Neill et al., 2013). For gluco(lipo)toxicity studies, cells were exposed to 33 mM glucose and/or 0.5 mM BSA-conjugated palmitate for 48 hr.

Statistical Analyses

Data normality was assessed using the D'Agostino Pearson omnibus test. Pairwise comparisons were performed using paired or unpaired Student's t test. Interactions between multiple treatments were determined using one-way or two-way ANOVA (adjusted for repeated-measures as necessary), followed by pairwise comparisons with Bonferonni's or Tukey's posthoc tests. Analyses were conducted using R (R Project), Graphpad Prism 6.0 (Graphpad Software), IgorPro (Wavemetrics), and MATLAB (Mathworks), and results deemed significant at $p < 0.05$. Unless otherwise stated, data are presented as the mean ± SEM.

SUPPLEMENTAL INFORMATION

Supplemental Information includes seven figures, six movies, and Supplemental Experimental Procedures and can be found with this article online at http://dx.doi.org/10.1016/j.cmet.2016.06.020.

AUTHOR CONTRIBUTIONS

N.R.J., G.A.R., and D.J.H. conceived and designed the experiments. N.R.J., R.K.M., E.H., M.P.P., F.S., and D.J.H. conducted the experiments. J.F. provided reagents and intellectual input. L.P., P.M., M.B., D.B., and E.B. isolated and provided human islet of Langerhans. P.D., M.W., J.B., and D.T. designed, synthesized, and provided chemical reagents. N.R.J., G.A.R., and D.J.H. wrote the paper with input from all the authors.

ACKNOWLEDGMENTS

N.R.J. was supported by a Diabetes UK RW and JM Collins Studentship (12/0004601). J.B. was supported by a European Foundation for the Study of Diabetes (EFSD) Albert Renold Young Scientist Fellowship and a Studienstiftung des deutschen Volkes PhD Studentship. D.T. was supported by an Advanced Grant from the European Research Commission (268795). G.A.R. was supported by Wellcome Trust Senior Investigator (WT098424AIA) and Royal Society Wolfson Research Merit Awards, and by MRC Programme (MR/J0003042/1), Biological and Biotechnology Research Council (BB/J015873/1), and Diabetes UK Project (11/0004210) grants. G.A.R. and M.W. acknowledge COST Action TD1304 Zinc-Net. D.J.H. was supported by Diabetes UK R.D. Lawrence (12/0004431), EFSD/Novo Nordisk Rising Star and Birmingham Fellowships, a Wellcome Trust Institutional Support Award, and an MRC Project Grant (MR/N00275X/1) with G.A.R. D.J.H and G.A.R. were supported by Imperial Confidence in Concept (ICiC) Grants. J.F. was supported by an MRC Programme grant (MR/L02036X/1). L.P. provided human islets through collaboration with the Diabetes Research Institute, IRCCS San Raffaele Scientific Institute (Milan), within the European islet distribution program for basic research supported by JDRF (1-RSC-2014-90-I-X). P.M. and M.B. were supported by the Innovative Medicine Initiative Joint Undertaking under grant agreement no. 155005 (IMIDIA), resources of which are composed of financial contribution from the European Union's Seventh Framework Programme (FP7/2007-2013) and EFPIA companies in kind contribution, and by the Italian Ministry of University and Research (PRIN 2010-2012). D.B. and E.B. provided human islets through the European Consortium for Islet Transplantation sponsored by JDRF (1-RSC-2014-100-I-X). We are grateful to Drs. Richard K.P. Benninger (University of Colorado Denver), Francois Molino (Université Montpellier II), and Stephen Rothery (FILM Facility, Imperial College London) for useful discussion. The authors have no conflicts of interest to disclose.

Received: December 16, 2015
Revised: May 17, 2016
Accepted: June 23, 2016
Published: July 21, 2016

REFERENCES

Ammälä, C., Larsson, O., Berggren, P.-O., Bokvist, K., Juntti-Berggren, L., Kindmark, H., and Rorsman, P. (1991). Inositol trisphosphate-dependent periodic activation of a Ca($^{2+}$)-activated K$^+$ conductance in glucose-stimulated pancreatic β-cells. Nature *353*, 849–852.

Andresen, V., Alexander, S., Heupel, W.M., Hirschberg, M., Hoffman, R.M., and Friedl, P. (2009). Infrared multiphoton microscopy: subcellular-resolved deep tissue imaging. Curr. Opin. Biotechnol. *20*, 54–62.

Benninger, R.K., and Piston, D.W. (2014). Cellular communication and heterogeneity in pancreatic islet insulin secretion dynamics. Trends Endocrinol. Metab. *25*, 399–406.

Benninger, R.K., Zhang, M., Head, W.S., Satin, L.S., and Piston, D.W. (2008). Gap junction coupling and calcium waves in the pancreatic islet. Biophys. J. *95*, 5048–5061.

Benninger, R.K., Hutchens, T., Head, W.S., McCaughey, M.J., Zhang, M., Le Marchand, S.J., Satin, L.S., and Piston, D.W. (2014). Intrinsic islet heterogeneity and gap junction coupling determine spatiotemporal Ca^{2+} wave dynamics. Biophys. J. *107*, 2723–2733.

Bonifazi, P., Goldin, M., Picardo, M.A., Jorquera, I., Cattani, A., Bianconi, G., Represa, A., Ben-Ari, Y., and Cossart, R. (2009). GABAergic hub neurons orchestrate synchrony in developing hippocampal networks. Science *326*, 1419–1424.

Bosco, D., Armanet, M., Morel, P., Niclauss, N., Sgroi, A., Muller, Y.D., Giovannoni, L., Parnaud, G., and Berney, T. (2010). Unique arrangement of alpha- and beta-cells in human islets of Langerhans. Diabetes *59*, 1202–1210.

Broichhagen, J., Schönberger, M., Cork, S.C., Frank, J.A., Marchetti, P., Bugliani, M., Shapiro, A.M.J., Trapp, S., Rutter, G.A., Hodson, D.J., and Trauner, D. (2014). Optical control of insulin release using a photoswitchable sulfonylurea. Nat. Commun. *5*, 5116.

Broichhagen, J., Frank, J.A., and Trauner, D. (2015). A roadmap to success in photopharmacology. Acc. Chem. Res. *48*, 1947–1960.

Bullmore, E., and Sporns, O. (2009). Complex brain networks: graph theoretical analysis of structural and functional systems. Nat. Rev. Neurosci. *10*, 186–198.

Desarménien, M.G., Jourdan, C., Toutain, B., Vessières, E., Hormuzdi, S.G., and Guérineau, N.C. (2013). Gap junction signalling is a stress-regulated component of adrenal neuroendocrine stimulus-secretion coupling in vivo. Nat. Commun. *4*, 2938.

Farnsworth, N.L., and Benninger, R.K. (2014). New insights into the role of connexins in pancreatic islet function and diabetes. FEBS Lett. *588*, 1278–1287.

Farnsworth, N.L., Hemmati, A., Pozzoli, M., and Benninger, R.K. (2014). Fluorescence recovery after photobleaching reveals regulation and distribution of connexin36 gap junction coupling within mouse islets of Langerhans. J. Physiol. *592*, 4431–4446.

Farnsworth, N.L., Walter, R.L., Hemmati, A., Westacott, M.J., and Benninger, R.K. (2015). Low Level Pro-Inflammatory Cytokines Decrease Connexin36 Gap Junction Coupling in Mouse and Human Islets through Nitric Oxide Mediated Protein Kinase Cδ. J. Biol. Chem. *291*, 3184–3196.

Fonseca, S.G., Gromada, J., and Urano, F. (2011). Endoplasmic reticulum stress and pancreatic β-cell death. Trends Endocrinol. Metab. *22*, 266–274.

Fujimoto, K., Hanson, P.T., Tran, H., Ford, E.L., Han, Z., Johnson, J.D., Schmidt, R.E., Green, K.G., Wice, B.M., and Polonsky, K.S. (2009). Autophagy regulates pancreatic beta cell death in response to Pdx1 deficiency and nutrient deprivation. J. Biol. Chem. *284*, 27664–27673.

Gerdes, J.M., Christou-Savina, S., Xiong, Y., Moede, T., Moruzzi, N., Karlsson-Edlund, P., Leibiger, B., Leibiger, I.B., Östenson, C.G., Beales, P.L., and Berggren, P.O. (2014). Ciliary dysfunction impairs beta-cell insulin secretion and promotes development of type 2 diabetes in rodents. Nat. Commun. *5*, 5308.

Head, W.S., Orseth, M.L., Nunemaker, C.S., Satin, L.S., Piston, D.W., and Benninger, R.K. (2012). Connexin-36 gap junctions regulate in vivo first- and second-phase insulin secretion dynamics and glucose tolerance in the conscious mouse. Diabetes *61*, 1700–1707.

Hodson, D.J., Molino, F., Fontanaud, P., Bonnefont, X., and Mollard, P. (2010). Investigating and modelling pituitary endocrine network function. J. Neuroendocrinol. *22*, 1217–1225.

Hodson, D.J., Schaeffer, M., Romanò, N., Fontanaud, P., Lafont, C., Birkenstock, J., Molino, F., Christian, H., Lockey, J., Carmignac, D., et al. (2012). Existence of long-lasting experience-dependent plasticity in endocrine cell networks. Nat. Commun. *3*, 605.

Hodson, D.J., Mitchell, R.K., Bellomo, E.A., Sun, G., Vinet, L., Meda, P., Li, D., Li, W.H., Bugliani, M., Marchetti, P., et al. (2013). Lipotoxicity disrupts incretin-regulated human β cell connectivity. J. Clin. Invest. *123*, 4182–4194.

Hraha, T.H., Westacott, M.J., Pozzoli, M., Notary, A.M., McClatchey, P.M., and Benninger, R.K. (2014). Phase transitions in the multi-cellular regulatory behavior of pancreatic islet excitability. PLoS Comput. Biol. *10*, e1003819.

Katsuta, H., Aguayo-Mazzucato, C., Katsuta, R., Akashi, T., Hollister-Lock, J., Sharma, A.J., Bonner-Weir, S., and Weir, G.C. (2012). Subpopulations of GFP-marked mouse pancreatic β-cells differ in size, granularity, and insulin secretion. Endocrinology *153*, 5180–5187.

Kiekens, R., In't Veld, P., Mahler, T., Schuit, F., Van De Winkel, M., and Pipeleers, D. (1992). Differences in glucose recognition by individual rat pancreatic B cells are associated with intercellular differences in glucose-induced biosynthetic activity. J. Clin. Invest. *89*, 117–125.

Konstantinova, I., Nikolova, G., Ohara-Imaizumi, M., Meda, P., Kucera, T., Zarbalis, K., Wurst, W., Nagamatsu, S., and Lammert, E. (2007). EphA-Ephrin-A-mediated beta cell communication regulates insulin secretion from pancreatic islets. Cell *129*, 359–370.

Li, D., Chen, S., Bellomo, E.A., Tarasov, A.I., Kaut, C., Rutter, G.A., and Li, W.H. (2011). Imaging dynamic insulin release using a fluorescent zinc indicator for monitoring induced exocytotic release (ZIMIR). Proc. Natl. Acad. Sci. USA *108*, 21063–21068.

Luo, J., Deng, Z.-L., Luo, X., Tang, N., Song, W.-X., Chen, J., Sharff, K.A., Luu, H.H., Haydon, R.C., Kinzler, K.W., et al. (2007). A protocol for rapid generation of recombinant adenoviruses using the AdEasy system. Nat. Protoc. *2*, 1236–1247.

Maedler, K., Størling, J., Sturis, J., Zuellig, R.A., Spinas, G.A., Arkhammar, P.O., Mandrup-Poulsen, T., and Donath, M.Y. (2004). Glucose- and interleukin-1beta-induced beta-cell apoptosis requires Ca2+ influx and extracellular signal-regulated kinase (ERK) 1/2 activation and is prevented by a sulfonylurea receptor 1/inwardly rectifying K$^+$ channel 6.2 (SUR/Kir6.2) selective potassium channel opener in human islets. Diabetes *53*, 1706–1713.

Mattis, J., Tye, K.M., Ferenczi, E.A., Ramakrishnan, C., O'Shea, D.J., Prakash, R., Gunaydin, L.A., Hyun, M., Fenno, L.E., Gradinaru, V., et al. (2012). Principles for applying optogenetic tools derived from direct comparative analysis of microbial opsins. Nat. Methods *9*, 159–172.

Meda, P., Atwater, I., Gonçalves, A., Bangham, A., Orci, L., and Rojas, E. (1984). The topography of electrical synchrony among beta-cells in the mouse islet of Langerhans. Q. J. Exp. Physiol. *69*, 719–735.

Michau, A., Hodson, D.J., Fontanaud, P., Guillou, A., Espinosa-Carrasco, G., Molino, F., Peters, C.J., Robinson, I.C., Le Tissier, P., Mollard, P., et al. (2015). Metabolism regulates exposure of pancreatic islets to circulating molecules in vivo. Diabetes *65*, 463–475..

Nyman, L.R., Wells, K.S., Head, W.S., McCaughey, M., Ford, E., Brissova, M., Piston, D.W., and Powers, A.C. (2008). Real-time, multidimensional in vivo imaging used to investigate blood flow in mouse pancreatic islets. J. Clin. Invest. *118*, 3790–3797.

O'Neill, C.M., Lu, C., Corbin, K.L., Sharma, P.R., Dula, S.B., Carter, J.D., Ramadan, J.W., Xin, W., Lee, J.K., and Nunemaker, C.S. (2013). Circulating levels of IL-1B+IL-6 cause ER stress and dysfunction in islets from prediabetic male mice. Endocrinology *154*, 3077–3088.

Palti, Y., David, G.B., Lachov, E., Mida, Y.H., and Schatzberger, R. (1996). Islets of Langerhans generate wavelike electric activity modulated by glucose concentration. Diabetes *45*, 595–601.

Pancholi, J., Hodson, D.J., Jobe, K., Rutter, G.A., Goldup, S.M., and Watkinson, M. (2014). Biologically targeted probes for Zn^{2+}: a diversity oriented modular "click-SNAr-click" approach. Chem. Sci. (Camb.) *5*, 3528–3535.

Pfisterer, C., Gruia, A., and Fischer, S. (2009). The mechanism of photo-energy storage in the Halorhodopsin chloride pump. J. Biol. Chem. *284*, 13562–13569.

Porat, S., Weinberg-Corem, N., Tornovsky-Babaey, S., Schyr-Ben-Haroush, R., Hija, A., Stolovich-Rain, M., Dadon, D., Granot, Z., Ben-Hur, V., White, P., et al. (2011). Control of pancreatic β cell regeneration by glucose metabolism. Cell Metab. *13*, 440–449.

Pralong, W.F., Bartley, C., and Wollheim, C.B. (1990). Single islet beta-cell stimulation by nutrients: relationship between pyridine nucleotides, cytosolic Ca^{2+} and secretion. EMBO J. *9*, 53–60.

Proserpio, V., and Lonnberg, T. (2015). Single cell technologies are revolutionizing the approach to rare cells. Immunol. Cell Biol. *94*, 225–229.

Ravier, M.A., Güldenagel, M., Charollais, A., Gjinovci, A., Caille, D., Söhl, G., Wollheim, C.B., Willecke, K., Henquin, J.C., and Meda, P. (2005). Loss of connexin36 channels alters beta-cell coupling, islet synchronization of glucose-induced Ca^{2+} and insulin oscillations, and basal insulin release. Diabetes *54*, 1798–1807.

Reinbothe, T.M., Safi, F., Axelsson, A.S., Mollet, I.G., and Rosengren, A.H. (2014). Optogenetic control of insulin secretion in intact pancreatic islets with β-cell-specific expression of Channelrhodopsin-2. Islets *6*, e28095.

Rickgauer, J.P., and Tank, D.W. (2009). Two-photon excitation of channelrhodopsin-2 at saturation. Proc. Natl. Acad. Sci. USA *106*, 15025–15030.

Rodriguez-Diaz, R., Speier, S., Molano, R.D., Formoso, A., Gans, I., Abdulreda, M.H., Cabrera, O., Molina, J., Fachado, A., Ricordi, C., et al. (2012). Noninvasive in vivo model demonstrating the effects of autonomic innervation on pancreatic islet function. Proc. Natl. Acad. Sci. USA *109*, 21456–21461.

Roma, L.P., Duprez, J., and Jonas, J.C. (2015). Glucokinase activation is beneficial or toxic to cultured rat pancreatic islets depending on the prevailing glucose concentration. Am. J. Physiol. Endocrinol. Metab. *309*, E632–E639.

Rutter, G.A., Pullen, T.J., Hodson, D.J., and Martinez-Sanchez, A. (2015). Pancreatic β-cell identity, glucose sensing and the control of insulin secretion. Biochem. J. *466*, 203–218.

Salomon, D., and Meda, P. (1986). Heterogeneity and contact-dependent regulation of hormone secretion by individual B cells. Exp. Cell Res. *162*, 507–520.

Santos, R.M., Rosario, L.M., Nadal, A., Garcia-Sancho, J., Soria, B., and Valdeolmillos, M. (1991). Widespread synchronous $[Ca^{2+}]_i$ oscillations due to bursting electrical activity in single pancreatic islets. Pflugers Arch. *418*, 417–422.

Speier, S., Nyqvist, D., Cabrera, O., Yu, J., Molano, R.D., Pileggi, A., Moede, T., Köhler, M., Wilbertz, J., Leibiger, B., et al. (2008). Noninvasive in vivo imaging of pancreatic islet cell biology. Nat. Med. *14*, 574–578.

Squires, P.E., Persaud, S.J., Hauge-Evans, A.C., Gray, E., Ratcliff, H., and Jones, P.M. (2002). Co-ordinated $Ca^{(2+)}$-signalling within pancreatic islets: does beta-cell entrainment require a secreted messenger. Cell Calcium *31*, 209–219.

Stolovich-Rain, M., Enk, J., Vikesa, J., Nielsen, F.C., Saada, A., Glaser, B., and Dor, Y. (2015). Weaning triggers a maturation step of pancreatic β cells. Dev. Cell *32*, 535–545.

Stožer, A., Gosak, M., Dolenšek, J., Perc, M., Marhl, M., Rupnik, M.S., and Korošak, D. (2013). Functional connectivity in islets of Langerhans from mouse pancreas tissue slices. PLoS Comput. Biol. *9*, e1002923.

Subach, F.V., Patterson, G.H., Renz, M., Lippincott-Schwartz, J., and Verkhusha, V.V. (2010). Bright monomeric photoactivatable red fluorescent protein for two-color super-resolution sptPALM of live cells. J. Am. Chem. Soc. *132*, 6481–6491.

Szabat, M., Luciani, D.S., Piret, J.M., and Johnson, J.D. (2009). Maturation of adult beta-cells revealed using a Pdx1/insulin dual-reporter lentivirus. Endocrinology *150*, 1627–1635.

Tarasov, A.I., Semplici, F., Ravier, M.A., Bellomo, E.A., Pullen, T.J., Gilon, P., Sekler, I., Rizzuto, R., and Rutter, G.A. (2012). The mitochondrial Ca2+ uniporter MCU is essential for glucose-induced ATP increases in pancreatic β-cells. PLoS ONE *7*, e39722.

Taylor, B.L., Liu, F.F., and Sander, M. (2013). Nkx6.1 is essential for maintaining the functional state of pancreatic beta cells. Cell Rep. *4*, 1262–1275.

Thorens, B., Tarussio, D., Maestro, M.A., Rovira, M., Heikkilä, E., and Ferrer, J. (2015). Ins1(Cre) knock-in mice for beta cell-specific gene recombination. Diabetologia *58*, 558–565.

Varadi, A., Grant, A., McCormack, M., Nicolson, T., Magistri, M., Mitchell, K.J., Halestrap, A.P., Yuan, H., Schwappach, B., and Rutter, G.A. (2006). Intracellular ATP-sensitive K+ channels in mouse pancreatic beta cells: against a role in organelle cation homeostasis. Diabetologia *49*, 1567–1577.

Váró, G., Zimányi, L., Fan, X., Sun, L., Needleman, R., and Lanyi, J.K. (1995). Photocycle of halorhodopsin from Halobacterium salinarium. Biophys. J. *68*, 2062–2072.

Zhang, F., Wang, L.P., Brauner, M., Liewald, J.F., Kay, K., Watzke, N., Wood, P.G., Bamberg, E., Nagel, G., Gottschalk, A., and Deisseroth, K. (2007). Multimodal fast optical interrogation of neural circuitry. Nature *446*, 633–639.

Zhang, M., Fendler, B., Peercy, B., Goel, P., Bertram, R., Sherman, A., and Satin, L. (2008a). Long lasting synchronization of calcium oscillations by cholinergic stimulation in isolated pancreatic islets. Biophys. J. *95*, 4676–4688.

Zhang, Q., Galvanovskis, J., Abdulkader, F., Partridge, C.J., Gopel, S.O., Eliasson, L., and Rorsman, P. (2008b). Cell coupling in mouse pancreatic beta-cells measured in intact islets of Langerhans. Philos. Transact. A Math. Phys. Eng. Sci. *366*, 3503–3523.

Cell Metabolism, Volume *24*

Supplemental Information

Beta Cell Hubs Dictate

Pancreatic Islet Responses to Glucose

Natalie R. Johnston, Ryan K. Mitchell, Elizabeth Haythorne, Maria Paiva Pessoa, Francesca Semplici, Jorge Ferrer, Lorenzo Piemonti, Piero Marchetti, Marco Bugliani, Domenico Bosco, Ekaterine Berishvili, Philip Duncanson, Michael Watkinson, Johannes Broichhagen, Dirk Trauner, Guy A. Rutter, and David J. Hodson

SUPPLEMENTAL FIGURES

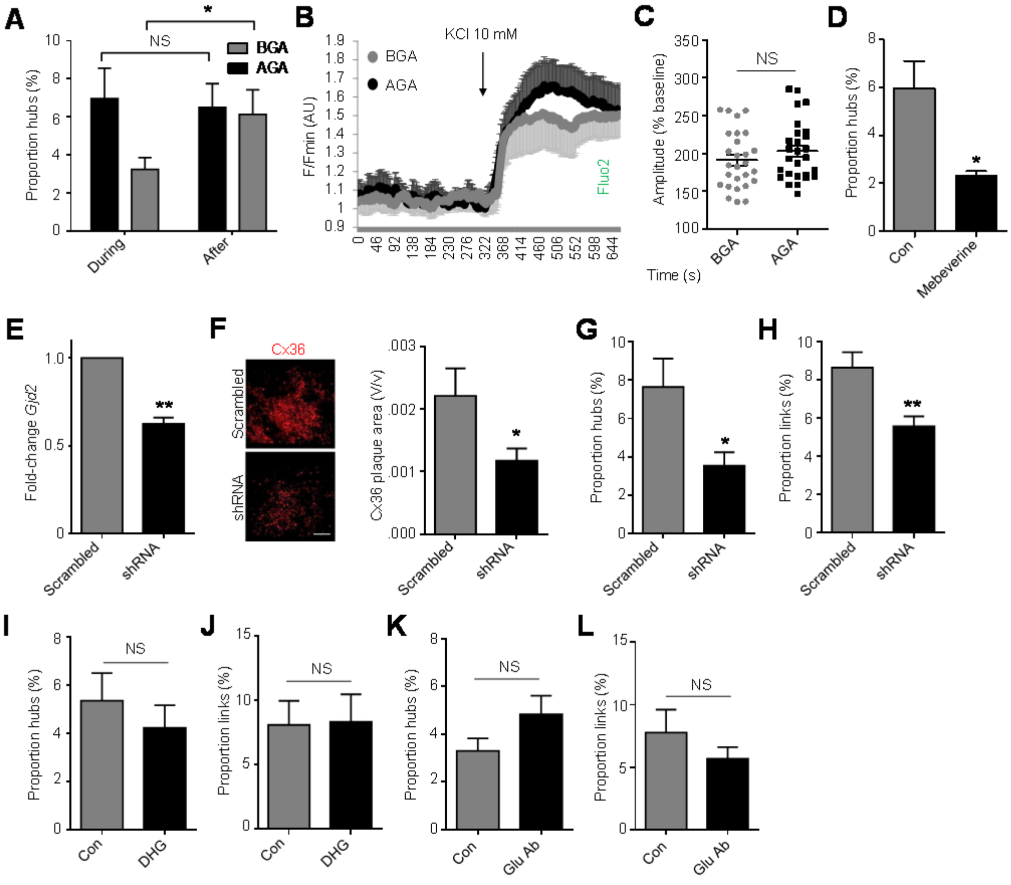

Figure S1. Gap junction inhibition with AGA is reversible and glucagon does not influence hub function. Related to Figure 1.

(A) The effects of 18-α-glycyrrhetinic acid (AGA; 20 µM) on hub number are reversible following washout (control is glycyrrhizic acid; BGA).

(B) Mean traces from a representative recording showing that AGA does not impair KCl-induced Ca^{2+} rises in dissociated single β cells *versus* BGA (20 µM) (*n* = 11 cells).

(C) As for (B), but summary data for all experiments (*n* = 27 cells from 3 animals).

(D) Mebeverine, another GJ blocker with a different mode of action, exerts similar effects to AGA (*n* = 5 recordings from 3 animals).

(E) Adenovirus harboring shRNA against *Gjd2* reduces expression by ~ 40% in whole islets (expected to be even higher at the surface) (*n* = 5 recordings from 6 animals) (scale bar, 25 µm).

(F) As for (E), but immunohistochemistry showing a large reduction in connexin-36 (Cx36) protein abundance following treatment with *Gjd2* shRNA (*n* = 7 islets from 3 animals).

(G-H) *Gjd2* knockdown decreases proportion of hubs (G) and correlated links (H) in mouse islets (*n* = 8 recordings from 6 animals).

(I-J) Hub function (I) and correlated population activity (J) is unaffected in the presence of 1 µM des-His[1]-[Glu[9]]-Glucagon (1-29) amide (DHG), a glucagon receptor antagonist (*n* = 6 recordings from 6 animals).

(K-L) As for (I-J), but in the presence of a mouse anti-glucagon antibody 1:1000 (*n* = 6 recordings from 6 animals).

Data are means ± SEM. **P<0.01, *P<0.05 and NS, non-significant.

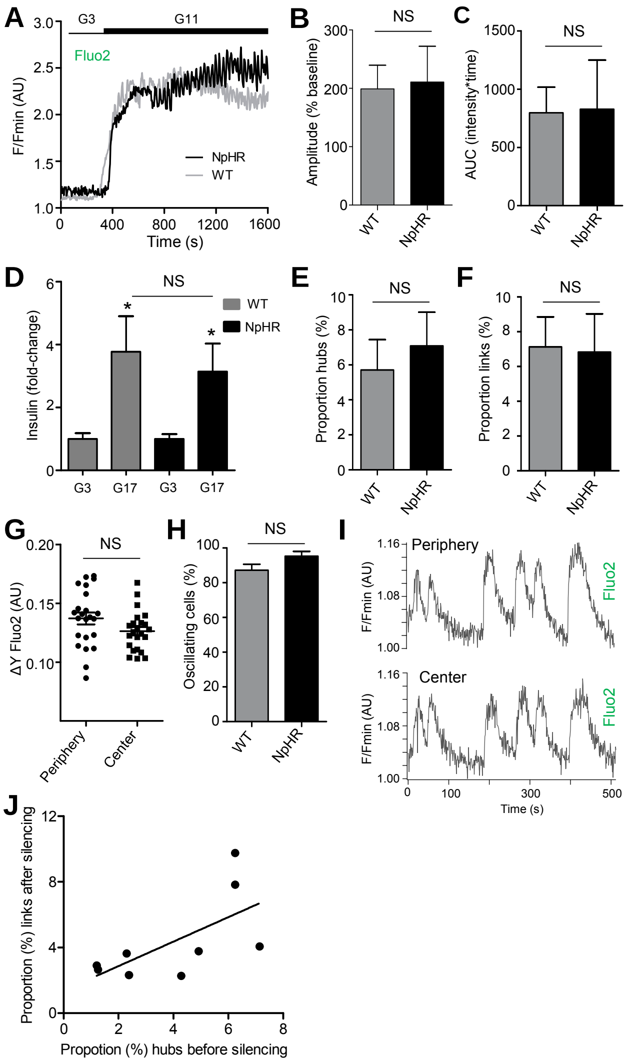

Figure S2. Ionic fluxes and insulin secretion in eNpHR3.0 islets, and hub number-silencing strength relationships. Related to Figure 4 and Figure 5.

(A) Glucose-stimulated intracellular Ca^{2+} rises are identical in wild-type (WT) and eNpHR3.0-expressing (NpHR) islets (mean of *n* = 6 recordings).

(B) Bar graph showing similar amplitude Ca^{2+} rises in WT and eNpHR3.0-expressing islets (*n* = 6 recordings).

(C) As for (B), but AUC.

(D) Glucose-stimulated insulin secretion *in vitro* is similar in WT and NpHR islets (NS, non-significant *versus* WT) (*n* = 4 animals).

(E) The number of hubs as a proportion of the imaged population is similar in WT and NpHR islets (*n* = 6 recordings).

(F) The proportion of correlated links (*i.e.* coordinated activity level) is similar in WT and NpHR islets (*n* = 6 recordings).

(G) No difference in the amplitude of Ca^{2+} rises is detected in cells located at the periphery or center of NpHR islets (*n* = 4 recordings).

(H) The proportion of cells displaying oscillatory behavior is similar in WT and NpHR islets (*n* = 6 recordings).

(I) Representative Ca^{2+} traces from the periphery and center of an NpHR islet.

(J) The strength of inhibition of islet Ca^{2+} responses (proportion (%) links) following targeting of an individual hub tends to be inversely correlated with the total number of these cells per islet before silencing (linear regression; R^2 = 0.42 and P<0.0601) (*n* = 9 recordings from 4 animals). That is, the non-silenced hubs can maintain some coordinated responsiveness.

For A-H, the signal attributable to membrane-bound EYFP was subtracted to allow more accurate comparison. Data are means ± SEM. *$P<0.05$ and NS, non-significant.

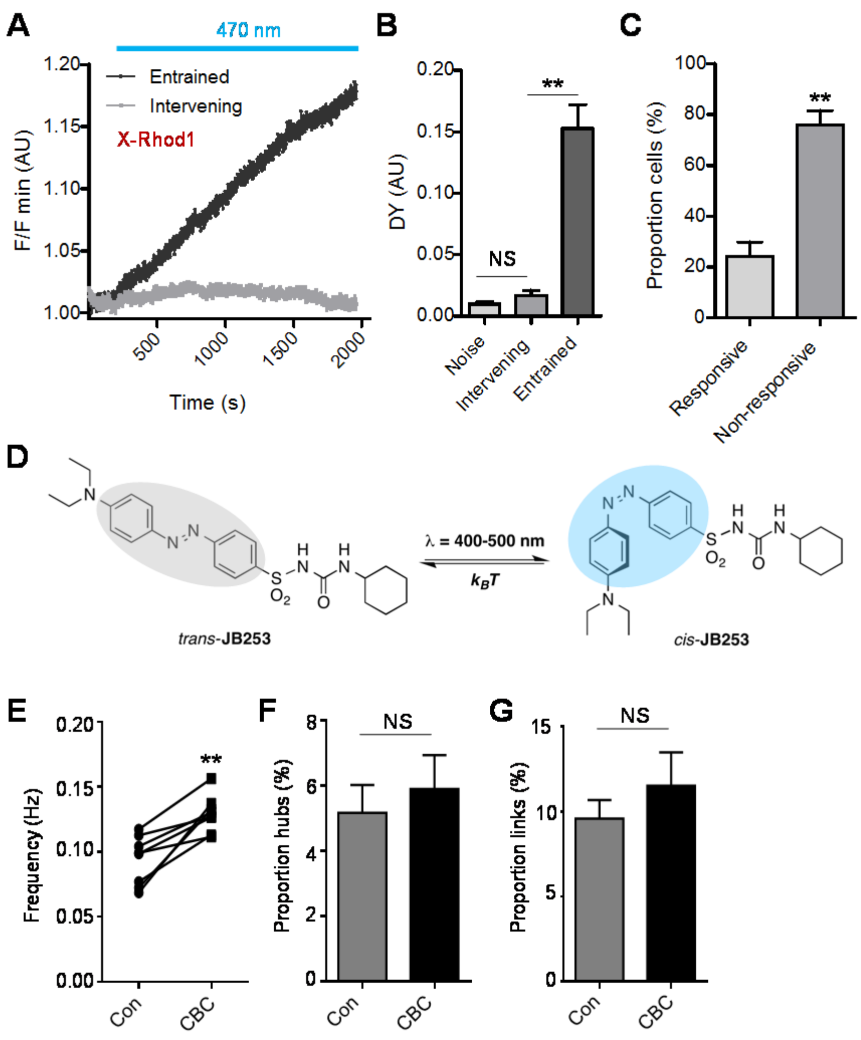

Figure S3. Photoactivated JB253 does not diffuse in the tissue and carbachol increases population frequency. Related to Figure 5.

(A) Mean intensity-over-time traces for X-Rhod1 in entrained and intervening (*i.e.* those located between the hub and follower) cells following targeted stimulation of a JB253-treated islet (n = 6 recordings from 4 animals).

(B) As for (A), but bar graph showing amplitude (DY) of Ca^{2+} rises in intervening and entrained cells.

(C) Only 20% of cells respond to targeted stimulation with Ca^{2+} rises, consistent with the number of followers linked to a hub (n = 6 recordings from 4 animals).

(D) Chemical structure of JB253 showing the *trans* (dark)- and *cis* (blue light)-isomers, the former possessing some activity in the dark due its photostationary state.

(E-G) The acetylcholinomimetic carbachol (CBC; 10 µM) increases population Ca^{2+}-spiking frequency (E), without altering the proportion of either hubs (F) or correlated links (G).

Data are means ± SEM. **P<0.01 and NS, non-significant.

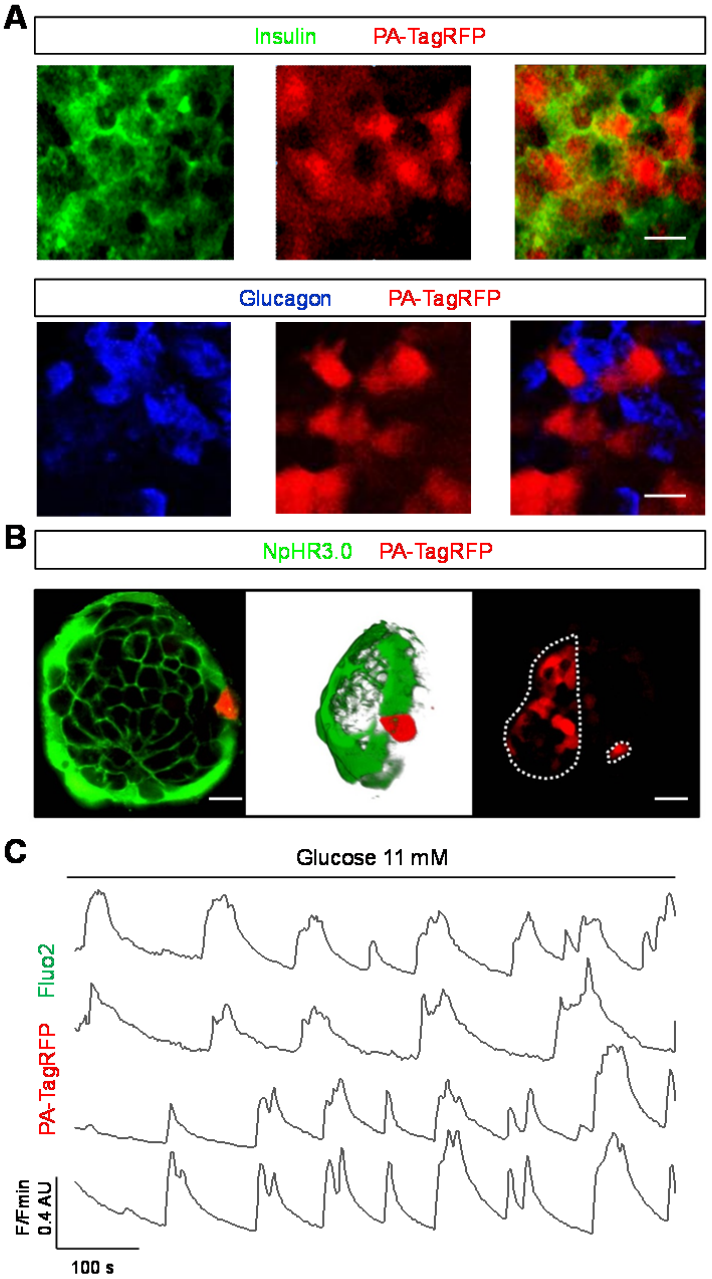

Figure S4. Single cell photoactivation of PA-TagRFP in intact islets. Related to Figure 6.

(A) PA-TagRFP is specifically expressed in β- but not α-cells, as assessed using immunostaining for insulin and glucagon, respectively (scale bar = 12.5 µm).

(B) Left panel: a single photopainted cell in a PA-TagRFP-expressing and fluo2-loaded islet ~ 40 µm from the surface (left) (63 x/1.4 NA objective). Middle panel: 3D opacity render of the same sample to show a single highlighted cell. Right panel: a single cell was targeted followed by half the islet (20x /0.8NA objective) (scale bar = 20 µm).

(C) Ca^{2+} oscillations are not perturbed by adenoviral-introduction of PA-TagRFP into islets (*n* = 3 recordings).

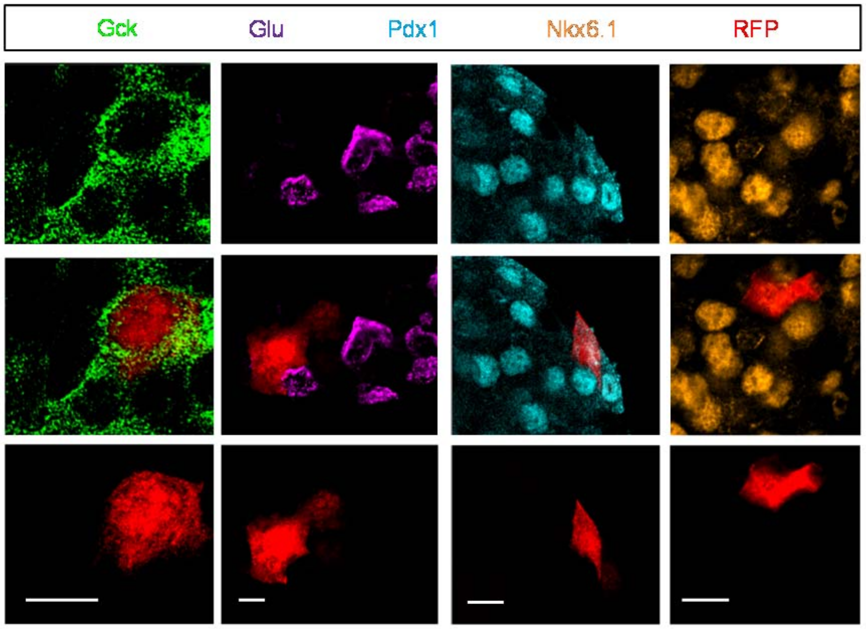

Figure S5. High-resolution snapshots of photopainted hubs. Related to Figure 6.

Hubs (RFP; red) express more glucokinase (Gck), less Pdx1 and less Nkx6.1 *versus* the rest of the population. No glucagon (Glu) expression is evident. Scale bar = 12.5 µm. Images captured in the intact islet preparation (*n* = 5-9 hubs from 3-4 animals) using a Zeiss LSM780 confocal microscope (63x/1.4NA objective).

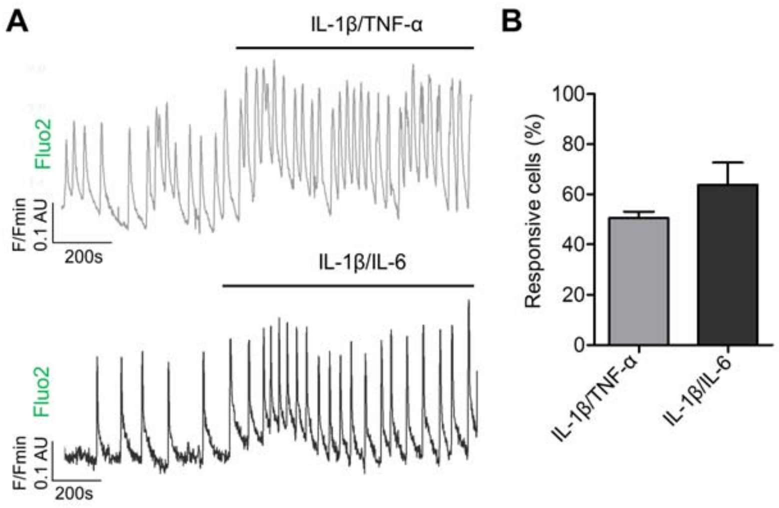

Figure S6. Acute effects of cytokine on β cell ionic fluxes. Related to Figure 7.

(A) Representative traces showing that application of either IL-1β/TNF-α or IL-1 β/IL-6 acutely upregulates Ca^{2+} spiking activity in islets.

(B) Bar graph showing that over half of the β cell population responds to acute cytokine exposure (*n* = 7-8 recordings from 4 animals) (186/368 cells for IL-1β/TNF-α; 174/279 cells for IL-1 β/IL-6).

Data are means ± SEM.

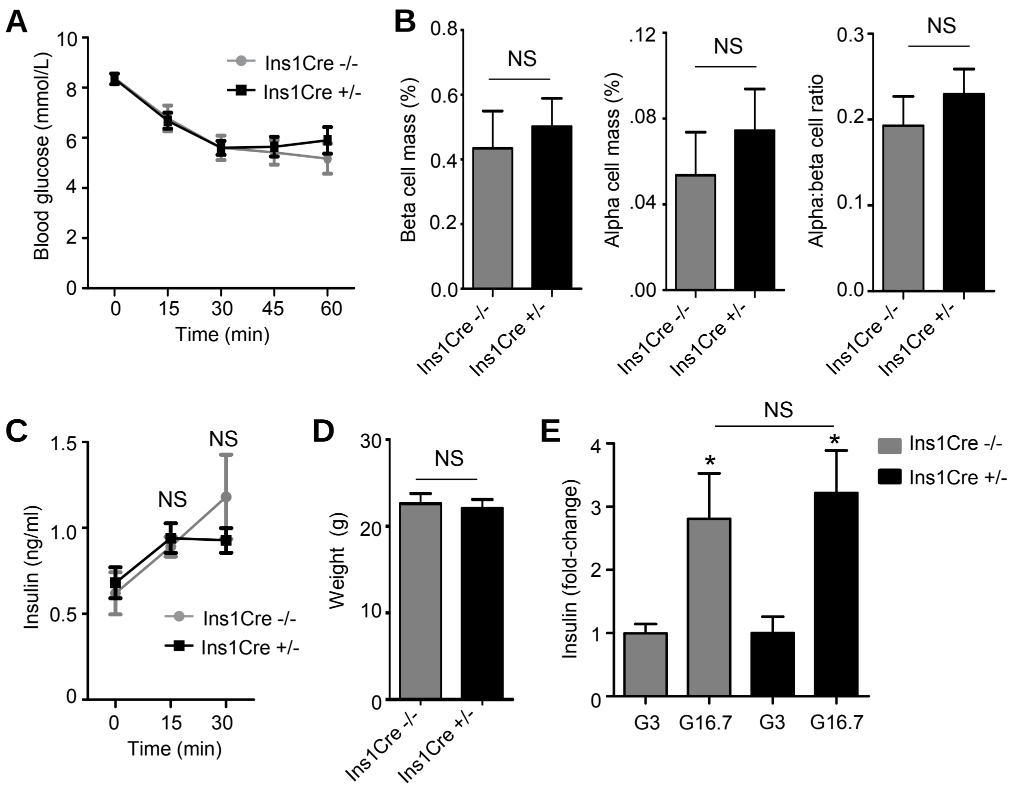

Figure S7. Phenotyping of Ins1Cre animals. Related to the Experimental Procedures.

(A) *Ins1Cre*$^{-/-}$ and *Ins1Cre*$^{+/-}$ animals display similar insulin tolerance (*n* = 4-6).

(B) β cell and alpha cell mass, as well as the α:β cell ratio are not significantly different in Ins1Cre$^{-/-}$ and Ins1Cre$^{+/-}$ animals (*n* = 3).

(C) *In vivo* insulin secretion is similar in Ins1Cre$^{-/-}$ and Ins1Cre$^{+/-}$ mice (*n* = 4-6).

(D) Body weights are similar in Ins1Cre$^{-/-}$ and Ins1Cre$^{+/-}$ mice (*n* = 4-6).

(E) Glucose-stimulated insulin secretion *in vitro* is similar in Ins1Cre$^{-/-}$ and Ins1Cre$^{+/-}$ islets (NS, non-significant *versus Ins1Cre*$^{-/-}$) (*n* = 4 animals).

Data are means ± SEM. *P<0.05 and NS, non-significant.

SUPPLEMENTAL MOVIE LEGENDS

Movie S1. Dynamics of hub function. Magnified timelapse recording of Ca^{2+} response in a wild-type islet showing a hub (circled in yellow) repetitively firing before the rest of the population. Islet was incubated in 11 mM glucose. Note the longer duty cycle. Playback rate 40 frames per second (fps). Related to Figure 1.

Movie S2. Optogenetic silencing of β cell function. Timelapse recording of Ca^{2+} responses in eNpHR3.0-expressing islets subjected to repeated silencing using a 561nm global laser (10 x objective). A Yokogawa Nipkow spinning disk head was used to rapidly scan a large field of view without introducing lag artefacts. A stable baseline from which to discriminate silencing from endogenous oscillatory activity was achieved by incubating islets in 100 µM tobultamide and 11 mM glucose. Movie has been cropped to display a single islet. Related to Figure 2.

Movie S3. Optogenetic targeting of β cells in a single islet. Timelapse recording of Ca^{2+} responses in two eNpHR3.0-expressing islets subjected to global silencing (left and right), before targeting of a single islet (right) (10 x objective). A stable baseline from which to discriminate silencing from endogenous oscillatory activity was achieved by incubating islets in 100 µM tobultamide and 11 mM glucose. Playback rate 40 frames per second (fps). Related to Figure 2.

Movie S4. Optogenetic targeting of β cells in an islet subregion. Timelapse recording of Ca^{2+} responses in an NpHR3.0-expressing islet subjected to global silencing (x1), before targeting (x2) of a cell triplet (right) (10 x objective). A stable baseline from which to discriminate silencing from endogenous oscillatory activity was achieved by incubating islets in 100 µM tobultamide and 11 mM glucose. Playback rate 40 frames per second (fps). Movie has been cropped to display a single islet. Related to Figure 2.

Movie S5: β cell population responses before hub silencing. $Beta$ cell population Ca2+ dynamics in eNpHR3.0-expressing islet before optogenetic silencing of a hub cell (20 x objective). Islet was incubated in 11 mM glucose. Playback rate 40 frames per second (fps). Related to Figure 5.

Movie S6: β cell population responses during hub silencing. Same islet as in Movie S5, but showing β cell population Ca2+ dynamics during optogenetic silencing of a hub cell (20 x objective). Islet was incubated in 11 mM glucose. Playback rate 40 frames per second (fps). Related to Figure 5.

SUPPLEMENTAL EXPERIMENTAL PROCEDURES

Animals, glucose/insulin tolerance testing and insulin measures

Mice were maintained in a specific pathogen-free (SPF) facility under a 12 h light–dark cycle with *ad libitum* access to water and standard laboratory diet. One copy of the floxed allele was sufficient to drive widespread eNpHR3.0-EYFP expression in β cells under the powerful ROSA26-targeted CAG promoter (*i.e. Ins1Cre*$^{+/-}$*;eNpHR3.0-EYFP*$^{fl/-}$, derived from an *Ins1Cre*$^{-/-}$*;eNpHR3.0-EYFP*$^{fl/fl}$ x *Ins1Cre*$^{+/-}$*;eNpHR3.0-EYFP*$^{-/-}$ breeding pair). Cre-mediated recombination efficiency was 94.3 ± 1.9 % in our hands, similar to that shown already (Thorens et al., 2014). To account for any effects that loss of an insulin allele may have on glucose tolerance, experiments were also performed in mice where eNpHR3.0 is activated on an Ins1Cre background (*i.e.* Ins1Cre$^{+/-}$;eNpHR3.0$^{fl/-}$ and *Ins1Cre*$^{+/-}$*;eNpHR3.0*$^{-/-}$ animals). Glucose and insulin tolerance tests were conducted using established protocols (Lemaire et al., 2009). Plasma insulin levels were determined at 0, 15 and 30 min following intraperitoneal injection of glucose (3 g/kg) using a Crystal Chem ultrasensitive ELISA. All procedures were regulated by the Home Office according to the Animals (Scientific Procedures) Act 1986 of the United Kingdom (PPL 70/7349).

Electrophysiology

β cells were identified morphologically and by depolarization of the membrane potential in response to 16.7 mM glucose. Extracellular solution was HEPES-buffered saline containing in mM: 135 NaCl, 5 KCl, 1 $MgCl_2$, 1 $CaCl_2$, 10 HEPES and 16.7 *D*-glucose (pH 7.4). For perforated patch, recording electrodes (5-10 MΩ resistance; borosilicate glass) were filled with a solution containing in mM: 140 KCl, 5 $MgCl_2$, 3.8 $CaCl_2$, 10 HEPES, 10 EGTA (pH 7.2) and 20–25 µg/ml amphotericin B (Beall et al., 2010). For whole-cell experiments, recording electrodes (5-10 MΩ resistance; borosilicate glass) were filled with a solution containing in mM: 140 KCl, 5 MgCl2, 3.8 $CaCl_2$, 10 HEPES, 10 EGTA (pH 7.2) and 3 ATP (Beall et al., 2010). These internal solutions contain similar levels of Cl^- to those used previously in β cells (Dadi et al., 2014) and for the study of eNpHR3.0 (Alfonsa et al., 2015; Song et al., 2013), and avoid liquid junction potential while maximising β cell action potential firing (Best, 2005). In perforated patch-clamp experiments, the resting membrane potential was recorded for 3 min before yellow light (λ = 572 nm) exposure for 3-5 min. In the voltage-clamp whole cell experiments, current was recorded in response to a 1 s pulse of yellow light.

Calcium and mitochondrial potential imaging

Islets were loaded with Fluo2 (Teflab) for 30-45 min before rapid capture of Ca^{2+} signals with cell resolution using a Yokogawa CSU22 Nipkow spinning disk coupled to a Zeiss Axiovert M200 and x10–x20/0.3–0.8NA objectives (Zeiss). By calculating the point-spread-function (PSF) of PS-Speck beads (100 nm; Invitrogen) for a 20x objective, this gives lateral and axial resolutions of 0.65 +/- 0.23 µm and 3.56 +/- 0.76 µm, respectively. Fluo2 was excited at λ = 491 nm using a DPSS laser (Cobalt) and emission detected at λ = 525/25 nm using a 16-bit EM-CCD (Hammamatsu). Ca^{2+} imaging using GCaMP6m was performed identically, except following 48 hr incubation with adenovirus (1.3 x 10^8 PFU/islet) harboring the cDNA for the genetically-encoded Ca^{2+} indicator (Vector Biolabs). Throughout, islets were maintained at 34-36 ^{0}C in a stainless steel chamber (Attofluor) and irrigated with HEPES-bicarbonate buffer containing in mM: 120 NaCl, 4.8 KCl, 24 $NaHCO_3$, 0.5 Na_2HPO_4, 5 HEPES, 2.5 $CaCl_2$, 1.2 $MgCl_2$ and 11 *D*-glucose. Single cell silencing in eNpHR3.0-expressing tissue was achieved using a λ = 589 nm laser (300 mW; CNI Laser) linked *via* a single-mode fibre optic to a custom-manufactured dichroic array, configured to deliver a diffraction-limited (~ 500 nm) spot (6.8-23.4 mW/mm^2) to the focal plane (Cairn Research). Single cell stimulation was performed using the photoswitchable sulfonylurea JB253 and a diffraction-limited λ = 470 nm laser.

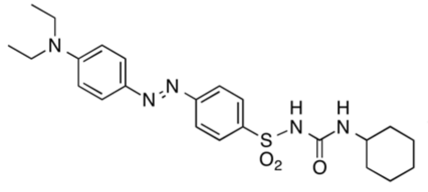

trans-**JB253**

JB253 was synthesized and used as previously described (Broichhagen et al., 2014), and combined with the Ca^{2+} indicator X-Rhod1 (excitation λ = 561 nm, emission λ = 630/75 nm) to avoid spectral overlap. JB253 occupies a photostationary state that results in ~10-20% K_{ATP} channel block (Broichhagen et al., 2014), a pre-requisite for proper propagation of signals *via* gap junctions (Zhang et al., 2008). However, following exposure to blue light, JB253 undergoes isomerization *via* its azobenzene photoresponsive unit, subtly changing molecule conformation at SUR1 to allow ~70% K_{ATP} channel closure (Broichhagen et al., 2014). Mitochondrial potential was monitored using 20 nM tetramethylrhodamine ethyl ester (TMRE) and excitation and emission at λ = 561 nm and λ = 630/75 nm, respectively (Hodson et al., 2014). Drugs were applied *via* the perfusion system at the indicated times and concentration.

Recordings were only included in subsequent analyses if: 1) the targeted cell itself responded with an increase/decrease in Ca^{2+} levels; and 2) silenced cells recovered from inhibition with resumption of Ca^{2+}-spiking activity. The overexpressed EYFP is unlikely to interfere with recordings of cytoplasmic Ca^{2+}, since the former is membrane-localized, and the amplitude of Ca^{2+} rises and AUC in response to elevated glucose was identical in wild-type and eNpHR3.0 islets, as shown in Figure S2. In all cases, care was taken to prevent artefacts due to phototoxicity/photodamage, for example by using multifocal microscopy to decrease pixel dwell time, employing highly-sensitive EM-CCD detectors, reducing laser power to the minimum required for an adequate signal-to-noise ratio (SNR), and limiting imaging duration. Experiments showing signs of phototoxicity (*i.e.* large and sustained increases/decreases in intracellular Ca^{2+} concentration, excessive photobleaching etc.) were discarded.

shRNA-silencing of connexin-36

Adenoviral particles carrying an shRNA expression construct against mouse *Gjd2* were acquired from Vector Biolabs and applied to islets at 7 x 10^9 PFU/islet for 24 hrs. Transduction was confirmed using GFP expression, as well as real-time PCR and immunohistochemistry for *Gjd2/*Cx36. In all cases, scrambled shRNA was used as control.

Immunohistochemistry

Confocal imaging was performed using a Zeiss LSM 780 Meta Confocal (x20-x63/0.8-1.4 NA objectives) configured with GaAsP spectral detectors set to collect emitted signals at 500-550 nm (Alexa Fluor-488), 561-597nm (Alexa Fluor-568) and 642-701nm (Alexa Fluor-633). The fraction area occupied by insulin, glucokinase (Gck), Pdx1, Tomm20, Nkx6.1, SERCA2 and Pdi was calculated using the threshold-corrected image (threshold plugin for ImageJ), and levels compared between photopainted cells and the remainder of the population. Mitochondrial morphology was assessed by measuring sphericity (Volocity) and length (BoneJ for Fiji) (Doube et al., 2010). To produce confocal superresolution images, the image was Nyquist-sampled in a 54 nm x 54 nm x 160 nm voxel, before iterative offline deconvolution using PSF algorithms embedded in Huygens software (Scientific Volume Imaging). The primary antibodies used were: guinea pig anti-insulin 1:200 (DAKO), mouse anti-glucagon 1:1000 (Sigma), rabbit anti-Gck 1:50 (Santa-Cruz), rabbit anti-Pdx1 1:50 (Rafiq, 2000), goat anti-Pdx1 1:2500 (Abcam), mouse anti-neurogenin 3 (Ngn3) 1:1000 (DSHB Hybridoma Product F25A1B3), rabbit anti-Tomm20 1:250 (Santa Cruz Biotechnology), rabbit anti-Nkx6.1 1:250 (Sigma), rabbit anti-SERCA2 1:250 (Alomone Labs), rabbit anti-Pdi 1:200 (Cell Signaling Technology) and rabbit anti-Cx36 1:50 (Zymed).The secondary antibodies used were Alexa Fluor-488, -568 and -633 (1:500) (Invitrogen). To live image mitochondria, islets were incubated in 100 nM MitoTracker Red FM (Invitrogen) for 30 min

β and α cell mass

β and α cell mass were measured using wax-embedded tissue slices taken at 25 µm intervals throughout the entire pancreas, and stained with guinea pig anti-insulin 1:200 (DAKO; Alexa Fluor-488 secondary) and rabbit anti-glucagon 1:100 (Santa-Cruz; Alexa Fluor-568 secondary) antibodies

Necrosis and apoptosis assays

Islets were incubated in 3 µM calcein AM (Invitrogen) and 2.5 µM propidium iodide (PI) (Sigma-Aldrich) for 15 min before detection of absorbance/emission at 491/525 nm and 561/620 nm, respectively. The islet area occupied by dead cells (PI) was expressed *versus* that occupied by live (calcein) and dead cells (V/v). Apoptosis was assessed using a DeadEnd Fluorimetric TUNEL System staining kit (Promega) according to the manufacturer's instructions and absorbance/emission detected at 491/525nm. The proportion of apoptotic β cells was expressed as a fraction area *versus* non-apoptotic cell mass (V/v).

Photopainting

Photopainting was performed *post facto* following fMCI and correlation analysis by bounding the identified cell(s) with a region of interest (ROI) and scanning the area with a λ = 405 nm diode laser (x20/0.8NA objective).

Correlation, similarity analyses and polar coordinates

Endocrine cells display Ca^{2+} spikes with complex frequencies, amplitudes and inter-event intervals, rendering algorithms based on spike detection unreliable. Thus, binarization was instead employed where cells are considered to be either 'ON' or 'OFF' at a given time point, depending on whether they are above a threshold value (20%; to account for the signal-to-noise ratio). The resulting binarized traces were subjected to correlation analyses based upon observations of cell synchrony using:

$$C_{ij} = \frac{T_{ij}}{\sqrt{T_i T_j}}$$

Where C is a correlation coefficient between -1 to +1, T_{ij} is the total time spent in the coactive state between node i and j, and T_i and T_i represent the time spent in the active phase for each individual node. To identify cell pairs correlated (*i.e.* linked) with a higher-than-chance probability, significance ($P<0.01$) of interactions was computed using a non-deterministic Monte-Carlo statistic *versus* the permuted dataset (> 9,999 iterations). Monte-Carlo analyses use computationally-intensive random sampling algorithms to model uncertainty (*i.e.* how likely are we to reproduce a given wiring patterns simply due to chance?). Functional connectivity maps were then constructed based on the distribution of correlations (or links) (k) between cells (n), allowing visualization of the network. The resulting link-probability distribution was best-fitted using the power-law function $f(x) = axk + o(xk)$ and cells occupying the 60-100% connectivity range (*i.e.* those possessing the majority of the links) defined as hubs. Power-law describes the relationship where a given variable changes proportionally to the power of the other. Such distributions are widely observed in physical and social systems and are typified by a long tail where the minority possess the majority (*e.g.* both wealth or scientific citations are power-law distributed). Goodness-of-fit was determined using a log-transformed linear regression model (R^2) and cross-checked against the non-linear minimization of the residual sum of squares (RSS) using heuristic methods (Nelder-Mead). To determine the stability of network topology, islets were considered 'before' and 'after' optogenetic silencing. The linear correlation between the resulting similarity matrices (*i.e.* how similar are the connection distribution/wiring patterns?) was then calculated using the Pearson R and significance considered against either: 1) a matrix with enforced dissimilarity (*i.e.* 'during' optogenetic silencing for each islet); or 2) a shuffled matrix (*i.e.* random permutation/shuffling for each islet). R values above and below 0 suggest positive and negative correlation, respectively. Polar coordinates (r,θ) were calculated from the Cartesian coordinates (x,y) using r (distance) = $\sqrt{(x^2 + y^2)}$ and $\theta = \tan^{-1}(y/x)$.

Measurements of insulin secretion from isolated islets

Insulin secretion was measured using static incubation of islets (batches of 6-8) for 30 min in HEPES-bicarbonate buffer at 37 °C containing the indicated glucose concentrations (da Silva Xavier et al., 2009). Insulin concentration in the supernatant was determined using a proprietary Homogeneous Time Resolved Fluorescence (HTRF)-based assay (Cisbio) according to the manufacturer's instructions.

Dynamic insulin secretion measures

Briefly, hubs were identified *a priori* using Ca^{2+} imaging before monitoring Zn^{2+} accumulation (excitation λ = 405 nm, emission λ = 450/50 nm) using an X-Light/Nikon Eclipse Ti-E spinning disk system in bypass mode and a x20/0.75 NA objective (Nikon). Cell silencing was performed with a user-defined ROI in Metamorph software and an $iLas^2$ photoactivation module (BioVision) coupled to a 585 nm laser (Coherent OBIS). Global silencing was performed using a 561 nm laser (Coherent OBIS). JP-107 is derived from click-S_NAR-click chemistry, contains a Zn^{2+}-chelating site and displays increases in fluorescence intensity due to accumulation of the ion over time (Pancholi et al., 2014). Thus, insulin secretion rate was calculated for each individual experiment by fitting a linear regression model. The mean slope value was then statistically compared for each condition examined (*i.e.* before and during laser exposure), and the P-value displayed on the corresponding graph. Negative rates are observed when insulin secretion is unable to surpass net Zn^{2+} dissolution from the probe in the presence of 1 μM EDTA (added to reduce background noise).

SUPPLEMENTAL REFERENCES

Alfonsa, H., Merricks, E.M., Codadu, N.K., Cunningham, M.O., Deisseroth, K., Racca, C., and Trevelyan, A.J. (2015). The contribution of raised intraneuronal chloride to epileptic network activity. J. Neurosci. *35*, 7715-7726.

Beall, C., Piipari, K., Al-Qassab, H., Smith, M.A., Parker, N., Carling, D., Viollet, B., Withers, D.J., and Ashford, M.L. (2010). Loss of AMP-activated protein kinase alpha2 subunit in mouse beta-cells impairs glucose-stimulated insulin secretion and inhibits their sensitivity to hypoglycaemia. Biochem. J. *429*, 323-333.

Best, L. (2005). Glucose-induced electrical activity in rat pancreatic beta-cells: dependence on intracellular chloride concentration. J. Physiol. *568*, 137-144.

Broichhagen, J., Schönberger, M., Cork, S.C., Frank, J.A., Marchetti, P., Bugliani, M., Shapiro, A.M.J., Trapp, S., Rutter, G.A., Hodson, D.J., et al. (2014). Optical control of insulin release using a photoswitchable sulfonylurea. Nat. Commun. *5*, 5116.

da Silva Xavier, G., Loder, M.K., McDonald, A., Tarasov, A.I., Carzaniga, R., Kronenberger, K., Barg, S., and Rutter, G.A. (2009). TCF7L2 regulates late events in insulin secretion from pancreatic islet beta-cells. Diabetes *58*, 894-905.

Dadi, P.K., Vierra, N.C., and Jacobson, D.A. (2014). Pancreatic beta-cell-specific ablation of TASK-1 channels augments glucose-stimulated calcium entry and insulin secretion, improving glucose tolerance. Endocrinology *155*, 3757-3768.

Doube, M., Kłosowski, M.M., Arganda-Carreras, I., Cordelières, F.P., Dougherty, R.P., Jackson, J.S., Schmid, B., Hutchinson, J.R., and Shefelbine, S.J. (2010). BoneJ: Free and extensible bone image analysis in ImageJ. Bone *47*, 1076-1079.

Hodson, D.J., Tarasov, A.I., Gimeno Brias, S., Mitchell, R.K., Johnston, N.R., Haghollahi, S., Cane, M.C., Bugliani, M., Marchetti, P., Bosco, D., et al. (2014). Incretin-modulated beta cell energetics in intact islets of Langerhans. Mol. Endocrinol. *28*, 860-871.

Lemaire, K., Ravier, M.A., Schraenen, A., Creemers, J.W., Van de Plas, R., Granvik, M., Van Lommel, L., Waelkens, E., Chimienti, F., Rutter, G.A., et al. (2009). Insulin crystallization depends on zinc transporter ZnT8 expression, but is not required for normal glucose homeostasis in mice. Proc. Natl. Acad. Sci. U. S. A. *106*, 14872-14877.

Pancholi, J., Hodson, D.J., Jobe, K., Rutter, G.A., Goldup, S.M., and Watkinson, M. (2014). Biologically targeted probes for Zn^{2+}: a diversity oriented modular “click-SNAr-click” approach. Chemical Science *5*, 3528-3535.

Rafiq, I. (2000). Glucose-stimulated Preproinsulin Gene Expression and Nuclear trans-Location of Pancreatic Duodenum Homeobox-1 Require Activation of Phosphatidylinositol 3-Kinase but Not p38 MAPK/SAPK2. J. Biol. Chem. *275*, 15977-15984.

Song, J., Sun, J., Moss, J., Wen, Z., Sun, G.J., Hsu, D., Zhong, C., Davoudi, H., Christian, K.M., Toni, N., et al. (2013). Parvalbumin interneurons mediate neuronal circuitry–neurogenesis coupling in the adult hippocampus. Nat. Neurosci. *16*, 1728-1730.

Thorens, B., Tarussio, D., Maestro, M.A., Rovira, M., Heikkila, E., and Ferrer, J. (2014). Ins1 knock-in mice for beta cell-specific gene recombination. Diabetologia *58*, 558-565.

Zhang, Q., Galvanovskis, J., Abdulkader, F., Partridge, C.J., Gopel, S.O., Eliasson, L., and Rorsman, P. (2008). Cell coupling in mouse pancreatic beta-cells measured in intact islets of Langerhans. Philos. Transact. A Math. Phys. Eng. Sci. *366*, 3503-3523.
